# Epigenetic and Mitochondrial Metabolic Dysfunction in Multiple Sclerosis: A Review of Herbal Drug Approaches and Current Clinical Trials

**DOI:** 10.1007/s12035-025-04868-8

**Published:** 2025-04-03

**Authors:** Ashwani, Anjali Sharma, Mayank Kumar Choudhary, Dalapathi Gugulothu, Deepti Pandita, Surajpal Verma, Lalitkumar K. Vora, Dharmendra Kumar Khatri, Debapriya Garabadu

**Affiliations:** 1 Delhi Pharmaceutical Sciences and Research University (DPSRU), New Delhi, 110017 India; 2 CDSCO, FDA Bhawan, New Delhi, India; 3 Department of Pharmacology, School of Basic and Applied Sciences, Central University of Punjab, Bathinda, 151401 India; 4 Department of Pharmacology, NIMS Institute of Pharmacy, NIMS University Rajasthan, Jaipur, 303121 India; 5 School of Pharmacy, Medical Biology Centre, Queen’S University Belfast, 97 Lisburn Road, Belfast, Northern Ireland BT9 7BL UK

**Keywords:** Epigenetic dysregulation, Mitochondria dysfunction, Multiple sclerosis, Herbal drugs, Clinical trials

## Abstract

Multiple sclerosis (MS) is a complex autoimmune disease characterised by inflammation, demyelination, and neurodegeneration within the central nervous system (CNS). While the exact causes remain unclear, recent research highlights the significant role of epigenetic modifications and mitochondrial dysfunction in the disease’s onset and progression. Epigenetic alterations, such as DNA methylation, histone modification, and microRNA regulation, influence gene expression without altering the DNA sequence, leading to immune dysregulation and inflammation. Similarly, mitochondrial dysfunction, marked by impaired oxidative phosphorylation, reduced adenosine triphosphate (ATP) production, and increased reactive oxygen species (ROS), contributes to neurodegeneration and impaired remyelination in MS. The growing interest in targeting these two interconnected mechanisms has opened new avenues for MS treatment. Herbal drugs, known for their multi-targeted effects, have shown potential in modulating epigenetic markers and enhancing mitochondrial function. Compounds such as resveratrol, curcumin, epigallocatechin-3-gallate (EGCG), quercetin, and omega-3 fatty acids demonstrate potential in regulating DNA methylation, histone deacetylation, and mitochondrial biogenesis. These natural agents offer dual-action therapies by reducing oxidative stress and inflammation while promoting neuronal survival and remyelination. This review explores the therapeutic potential of herbal drugs targeting epigenetic and mitochondrial pathways in MS, evaluating their mechanisms of action and highlighting their promise as novel therapeutic agents. While initial findings are encouraging, further research and clinical trials are required to validate the efficacy of these herbal treatments and fully understand their potential in slowing disease progression and improving patient outcomes in MS. Such exploration could pave the way for safer, multi-targeted therapies, offering new hope in the management of MS and other neurodegenerative diseases.

## Introduction

**Fig. 1:**
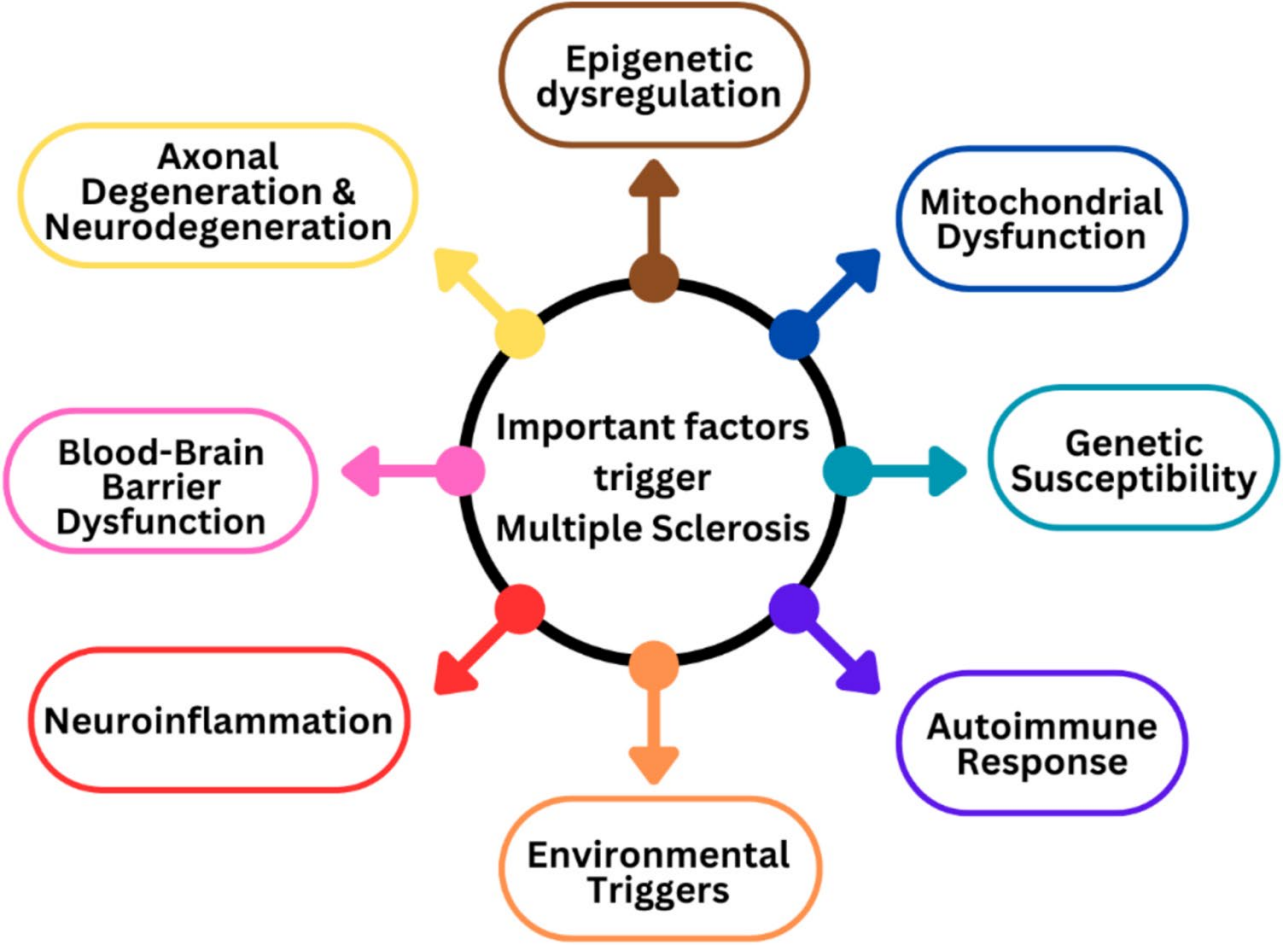
Depicting the important factors triggers the multiple sclerosis

Multiple sclerosis (MS) is a neurological disorder that has an impact on the central nervous system (CNS), including the brain and spinal cord, which can be chronic and possibly debilitating. It is categorised as an autoimmune illness since the body’s immune system unintentionally destroys its myelin, which serves as the protective sheath for CNS nerve fibres [1]. Perivascular lymphocytic invasion and macrophages, which cause the degeneration of myelin sheaths covering neurons, are the hallmarks of the disease. Initial inflammation is short and temporary where possible remyelination takes place. As a result, the disease’s early stages are marked by short episodes of neurological impairment that normally pass [2]. Chronic and severe neurodegeneration results from persistent and widespread microglial activation, which dominates the pathogenic alterations over time. As the clinical relationship develops or persists, the effect on the MS affected individual’s skills and day-to-day functioning progressively worsens. This may involve challenges with movement, coordination, cognitive abilities, and other facets of everyday living, which can result in an intensifying degree of impairment. Many other neurological symptoms might appear, such as visual loss, tingling or numbness, focal weakness, bladder or bowel incontinence, and cognitive failure. The particular symptoms change depending on exactly where the lesions occur. Evoked electrophysiological potentials, MRI abnormalities indicating the distribution of inflammatory lesions and axonal loss, intrathecal oligoclonal antibody synthesis, and cerebrospinal fluid examination by lumbar puncture are all examples of paraclinical investigations that reveal abnormalities [2, 3]. The research indicates that persistent neurodegeneration can also result from microglial activation; this condition is known as gliosis. Young people typically, initially encounter acute relapses as clinical symptoms, which are then followed by a steadily progressing course that within 10 to 15 years, results in permanent incapacity [4, 5]. A global survey indicated that 2.8 million individuals worldwide had MS as of September 11, 2020, and a new case of MS is discovered worldwide every 5 min, and nearly one million of them are American citizens [6]. According to estimates, there are 35.9 MS cases per 100,000 people worldwide, and rates have been rising since 2013. The mean age at diagnosis is 32 years, and the combined incidence rate among the 75 reporting nations is 2.1 cases per 100,000 people per year. In comparison to men, women have a twofold greater likelihood of having MS. There are still gaps in prevalence estimates, which highlights the need for more study and focus on comprehending and treating the MS [6]. Multiple risk factors can contribute to the complicated neurological condition MS. Along with other processes and causes, epigenetic and mitochondrial dysfunction play important roles in these. The important factors linked to MS are depicted in Fig. 1.

Epigenetic dysregulation: Epigenetic alterations modify the structure of DNA without changing the underlying genetic coding, affecting how genes are expressed. Epigenetic process dysregulation can result in aberrant gene expression, which may aid in the onset and development of MS [7].

Mitochondrial dysfunction: The energy-producing powerhouse of cells, mitochondria, can malfunction. The pathophysiology of MS may be influenced by mitochondrial dysfunction, which can result in reduced energy generation, oxidative stress, and poor cellular activity [8].

Genetic susceptibility: Although the precise origin of MS is still unclear, there is a hereditary component to the illness. There may be a hereditary predisposition to MS because several genetic variations have been associated with a higher risk of the disease [9].

Autoimmune response: MS is seen as an autoimmune illness in which the immune system inadvertently targets the central nervous system, particularly the myelin coating that protects nerve fibres. Neurological deficits, demyelination, and inflammation are the results of this immune-mediated onslaught [10].

Environmental triggers: It is thought that environmental factors can cause MS in those who are genetically predisposed to the disease. Infections, vitamin D insufficiency, smoking, and exposure to certain chemicals are a few possible reasons [11, 12].

Neuroinflammation: One of the distinguishing features of MS is CNS inflammation. Myelin breakdown and nerve injury are facilitated by the production of pro-inflammatory cytokines and other chemicals by activated immune cells [13].

Blood–brain barrier dysfunction: Under normal circumstances, the blood–brain barrier shields the brain from potentially dangerous compounds in the blood. The blood–brain barrier breaks down in MS, making it possible for immune cells to enter the brain and start the autoimmune reaction [14].

Axonal degeneration and neurodegeneration: Along with myelin loss, axonal degeneration also occurs in MS, impairing neuronal function permanently and accelerating the course of disability. MS may eventually cause neurodegeneration, which is characterised by the loss of neurons and brain mass and worsens impairment [15, 16].

While recent literature, such as Manna et al. (2024), provides a detailed insight of epigenetic changes, offers significant advancements by focusing on unique interconnections and novel therapeutic strategies. Advanced methodological techniques, such as CRISPR-dCas9 systems and multi-omics integration, are also involved in certain epigenetic changes affect immune responses and mitochondrial biogenesis in MS, offering a thorough and novel understanding of the disease. This study examines the consequences of mitochondrial dysfunction and epigenetic dysregulation in a natural therapy framework, highlighting their interrelated roles in MS [17]. These two mechanisms, which are becoming recognised as crucial components in the onset and progression of MS, are closely related to the pathophysiology of autoimmune illnesses. There is mounting evidence that mitochondrial dysfunction and epigenetic modifications impact immunological responses, neuroinflammation, and the degeneration of myelin and axons in the CNS, despite the fact that the exact aetiology of MS remains complex and multifaceted. For example, immunological and CNS cells exhibit abnormal DNA methylation, histone acetylation, and miRNA expression when exposed to ROS produced by malfunctioning mitochondria. Additionally, methyl donors such S-adenosylmethionine (SAM) are less readily available due to mitochondrial energy deficiencies, which has a direct effect on DNA and histone methylation. This manuscript, in contrast to previous research, offers a thorough examination of the distinct effects of epigenetic modifications, such as DNA methylation, histone modifications, and miRNA expression, on the four clinical courses of MS: relapsing–remitting MS (RRMS), secondary progressive MS (SPMS), primary progressive MS (PPMS), and progressive-relapsing MS (PRMS) [18]. The paper also discusses new treatment approaches that use natural substances like curcumin and resveratrol that target both epigenetic dysregulation and mitochondrial failure. This dual-targeting strategy is suggested as a new MS intervention. It highlights how metabolic intermediates like acetyl-CoA and SAM connect mitochondrial and epigenetic processes, providing information on the metabolic-epigenetic interactions in MS.

###  Role of Epigenetic Modifications and Mitochondrial Dysfunction in the Development and Progression of MS

DNA methylation, histone changes, and microRNA regulation are examples of epigenetic processes [19]. Immune cells, notably T cells and B cells, which are crucial in the autoimmune response, have shown these alterations in MS [18]. Gene expression that is involved in immune cell activation, regulation, and function can vary as a result of epigenetic modifications. Inappropriate immune responses against myelin proteins in the CNS and subsequent autoimmune attacks in MS may be caused by abnormal epigenetic patterns in immune cells [20]. Epigenetic changes connected to MS have been linked to environmental variables such as viral infections and vitamin D insufficiency. These environmental factors can start epigenetic alterations that turn on or off immune response-related genes, accelerating the progression of MS [21]. Epigenetic modifications can also influence genes associated with MS susceptibility. Genetic factors, combined with epigenetic changes, may increase the likelihood of developing the disease [22]. On the other side, the important organelles known as mitochondria are in charge of oxidative phosphorylation, which is the process by which ATP is produced. Cells of the immune system and the central nervous system have both been found to have mitochondrial dysfunction in MS [8]. Immunological cells with dysfunctional mitochondria may produce less energy, which affects their capacity to efficiently control immunological responses. This energy shortfall may cause the inflammation and immune cell activation seen in MS [23]. Reactive oxygen species (ROS) can be overproduced as a result of mitochondrial malfunction. Increased ROS levels cause oxidative stress, which damages cellular elements including myelin and axons that fuels the inflammation [24, 25]. Demyelination and axonal damage are indication of MS, and these may be caused by mitochondrial dysfunction inside CNS cells that stops the myelin from being repaired and maintained [8].

The pathophysiology of MS involves a complicated interaction between epigenetic alterations and mitochondrial dysfunction. Genes involved in metabolism, ROS generation, and mitochondrial function may express differently as a result of epigenetic modifications. On the other hand, epigenetic alterations can be impacted by mitochondrial malfunction via a variety of methods [26, 27]. Epigenetic dysregulation and mitochondrial dysfunction can have synergistic consequences that result in abnormal immunological responses, ongoing inflammation, and myelin degradation; all of these factors facilitate the progression of MS [22].

The study looks at possible natural therapeutic approaches that target both mitochondrial and epigenetic dysfunction, with an emphasis on how these interrelated mechanisms cause MS pathogenesis. The review’s investigation of this dual-action strategy attempts to provide focus on novel therapeutic approaches that may improve MS management and therapy, hence enhancing patient outcomes and delaying the course of the disease.

##  Epigenetic Modifications in Multiple Sclerosis

###  Epigenetic Modifications and Their Impact on Gene Expression Without Altering DNA Sequence

The study of the epigenome, a collection of chemical substances that control how the entire genome functions, is known as epigenomics. These substances attach to DNA and activate or deactivate particular genes within the genome, changing how the genome functions. As a result, the creation of proteins in some body cells may be controlled by the epigenome. “Marking” the genome refers to the process of epigenomic chemicals attaching to DNA and having an impact on how it functions [28]. While these markings do not change the DNA’s sequence, they do change how the DNA works. These changes may modify how genes are activated or inactivated, influencing several biological processes including development, differentiation, and responsiveness to environmental signals. Here are several significant epigenetic alterations and how they affect gene expression:

### DNA Methylation

**Fig. 2:**
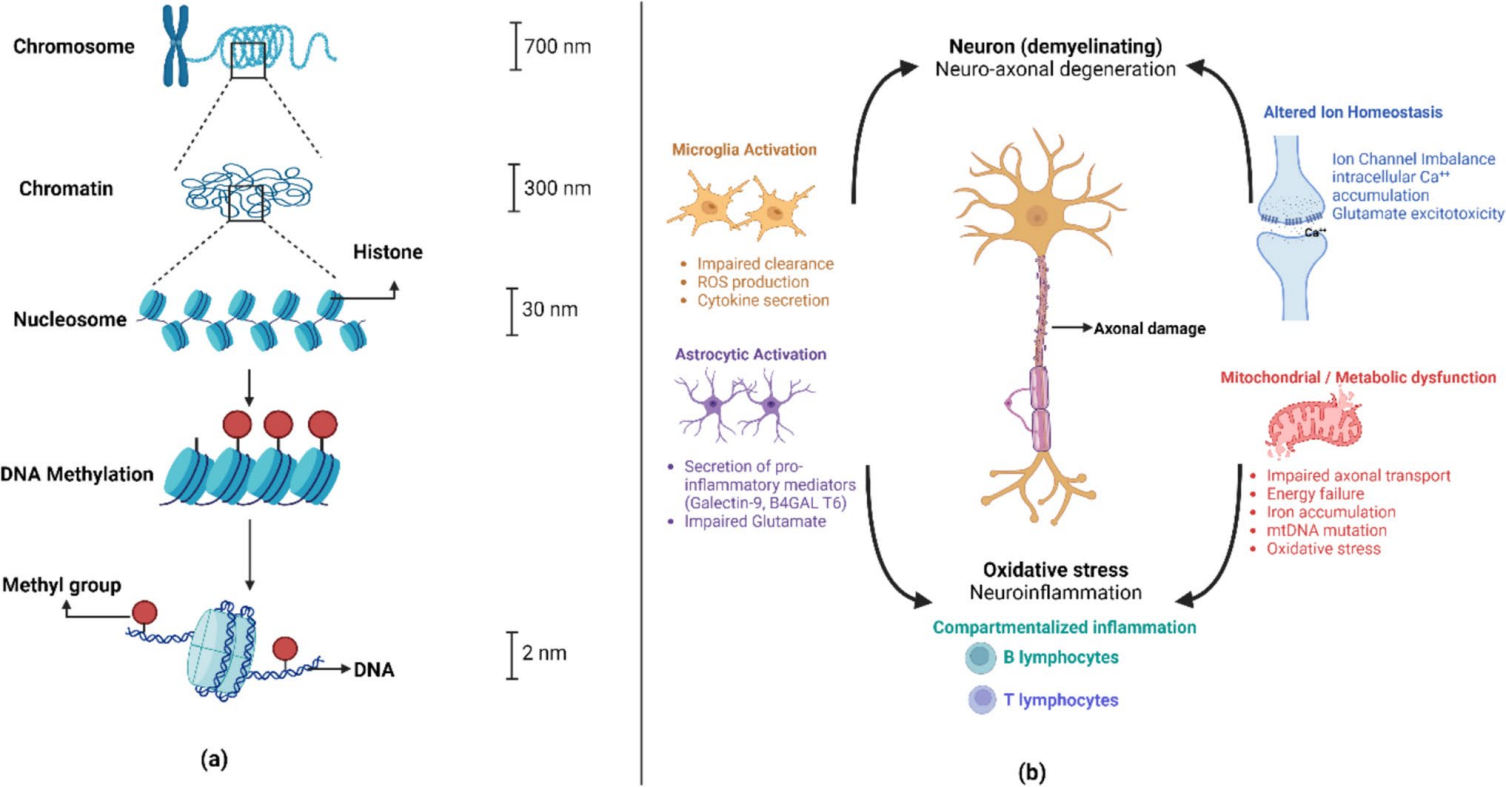
** a** Epigenetic processes regulate gene expression via histone modifications and DNA methylation, impacting DNA accessibility. **b** Mechanisms underlying progressive MS. In progressive MS, immune and CNS-resident cells release harmful mediators, perpetuating demyelination and neuro-axonal damage. Defective oligodendrocytes increase energy demands, while mitochondrial damage and ion imbalance exacerbate tissue injury, contributing to clinical symptoms

The cytosine nucleotide of DNA is modified in this process by the addition of a methyl group. DNA methylation frequently takes place at CpG sites (areas comprising a cytosine and a guanine). Gene silencing can result from increased DNA methylation in the promoter regions of genes because it prevents transcription factors and other regulatory proteins from binding, which reduces gene expression [28]. Epigenetic processes play a significant role in the complicated regulation of gene expression. DNA accessibility is impacted by histone complexes’ compacting of chromatin. This mechanism is tuned by DNA methylation and histone changes, which control gene expression. Genes are suppressed by cytosine methylation, especially at promoters. These methods work together and involve regulatory elements to modify DNA accessibility [29]. Their interaction, which affects the control of gene expression and cellular processes, is shown in Fig. 2a. Complex mechanisms resulting in persistent demyelination and neuro-axonal damage are part of the progressive MS development. Inflammatory mediators, reactive oxygen species, and iron species are released as a result of exposure to a hazardous microenvironment, which is partially mediated by immune cells (both peripheral and CNS-compartmentalised) and CNS-resident cells (microglia and astrocytes). This mechanism has a sizable impact on the continuous harm seen in progressive MS [30]. The structural and trophic support that is impaired because of myelinating oligodendrocytes that are not functioning properly increases the energy requirements for nerve-impulse conduction. A series of negative outcomes result from improper electrical activity being hampered by mitochondrial damage and disturbed ion homeostasis. Energy failure and oxidative stress within the damaged brain tissue are made worse by the existence of defective mitochondria, which are characterised by reduced axonal transport, susceptibility to oxidative damage, and mutations in mitochondrial DNA [31]. Additionally, an imbalance in ion channels, particularly too much glutamate, causes cytoplasmic calcium to build up and ultimately causes excitotoxicity. Collectively, these cascading alterations lead to the progressive character of MS, which causes the clinical symptoms and neurological deficits seen in affected people [32, 33]. Figure 2b provides a thorough explanation of these interrelated pathways that underlie the development of MS.

**Fig. 3:**
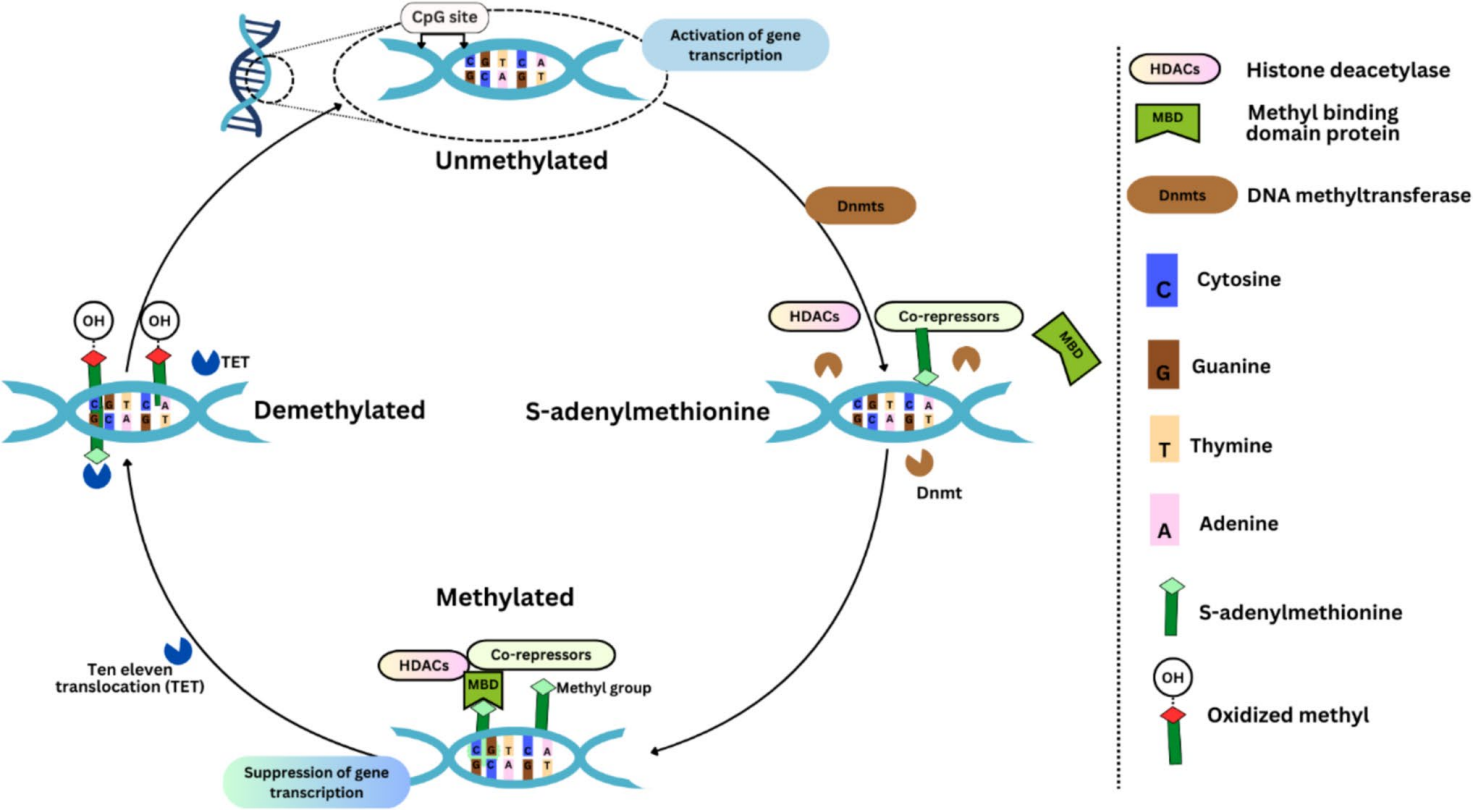
The regulatory mechanisms of DNA methylation in gene transcription involve complex interactions. DNA methyltransferases (Dnmts) use methyl groups provided by S-adenosyl methionine (SAM) to methylate the fifth carbon of cytosine, converting it to 5-methylcytosine (5mC). In mammals, this methylation typically occurs at CpG sites within gene promoter regions, leading to the repression of gene transcription and silencing by hindering the gene’s transcriptional activity. Conversely, ten-eleven translocation (TET) enzymes can oxidise 5mC to 5-hydroxymethylcytosine (5hmC), which facilitates DNA demethylation and allows transcription to resume [34]

DNA methyltransferases (Dnmts) are the primary regulators of this process, whereas the ten-eleven translocation (TET) family of enzymes reverses it. The functions of several Dnmts (Dnmt1, Dnmt2, and Dnmt3) in creating or preserving DNA methylation are explained in the text, along with the role that methyl-binding domain proteins (MBDs) play in the suppression of genes after methylation. It also emphasises how important these mechanisms are for immune control and the aetiology of MS. Dnmts use S-adenosyl methionine (SAM) as the methyl donor to accomplish DNA methylation. Dnmt3 starts de novo methylation, Dnmt2 targets tRNA, and Dnmt1 sustains methylation following replication. Histone deacetylases (HDACs) and co-repressors are enlisted in transcription suppression by MBD proteins like MeCP2. By transforming 5-methylcytosine (5mC) into 5-hydroxymethylcytosine (5hmC), the TET family promotes DNA demethylation. The regulatory mechanism of DNA methylation involving TET, 5hmc, SAM, and Dnmts is shown in Fig. 3. In MS, there has also been aberrant demethylation as well as methylation at these sites. An outline of these findings and their potential impact on the pathophysiology and risk of MS is given in Table 1.

**Table 1:** The substantial effects of methylation alterations on immunological regulation, neuronal survival, and gene expression in both MS patients and EAE models. The results highlight the ways that inflammation and MS pathogenesis are influenced by the hypermethylation or hypomethylation of important genes, providing possible biomarkers and therapeutic targets for intervention

Gene	methylation state	Expression	Research object	Effect of methylation changes in EAE mice and proposed effect in MS patients	References
SHP-1	Hypermethylation	Reduced	MS patients	Because the SHP-1 gene is hypermethylated, its expression is decreased, which raises leukocyte-mediated inflammation by preventing pro-inflammatory signalling pathways from being negatively regulated	[35]
PAD2	Demethylation	Enhanced	MS patients	The PAD2 gene becomes more expressed when it is demethylated, which raises the amount of PAD2 protein production. Because of the increased citrullination of myelin basic protein (MBP), this increase adds to myelin instability	[36]
GMN	Hypomethylation	Enhanced	MS patients	In normal-appearing white matter (NAWM), hypomethylation of the GMN gene may reduce neuronal survival and cause the generation of immunogenic peptides	[37]
BCL2L2	Hypermethylation	Reduced	MS patients	The control of neuronal and oligodendrocyte survival in NAWM may be impacted by BCL2L2 hypermethylation, which may exacerbate MS pathophysiology	[38]
AGHL, NDRG1, DNHD1, and others	Hypermethylation	Reduced	MS patients	Through cis-elements, transcription factor networks, and other biological processes, hypermethylation of these genes may impact CD8 + T cell activity and motility, hence raising vulnerability to multiple sclerosis	[39]
HLA-DRB5	No change	Enhanced	MS patients	The HLA-DRB5 gene’s DNA methylation in peripheral blood did not significantly alter. But HLA-DRB5-in particular, DRB5*0101-interacts with HLA-DRB1 to limit T cells that are specific to MBP	[40]
HLA-DRB1	Hypermethylation	Reduced	MS patients	The presentation of peptides produced from extracellular proteins is impacted by the hypermethylation of HLA-DRB1 in CD4 + T cells, which may influence the immunological response in multiple sclerosis	[41]
TET2	-	Reduced	MS patients	Abnormal methylation of target genes is connected to alterations in the methylation pattern of peripheral blood mononuclear cells (PBMCs) involving TET2, which raises the risk of MS	[42]
5hmC, Dnmt1, MeCP2	-	Enhanced	EAE mice	Brain-derived neurotrophic factor (BDNF) signalling is modulated by elevated levels of MeCP2 in EAE mice, which inhibits remyelination and myelin repair. This suggests a possible function for MeCP2 in MS by blocking BDNF-induced myelin repair	[43, 44]
IL-4/Foxp3	Hypermethylation	Reduced	EAE mice	The generation of Th1 cells, macrophages, and IFN-γ is reduced when the IL-4/Foxp3 locus is hypermethylated, which protects CD44 + encephalitogenic T cells against EAE	[45]
IFN-γ/IL-17a	Demethylation	Enhanced	EAE mice	The process of inflammation is initiated and naïve T helper cells are encouraged to differentiate into Th2 cells by demethylation of the IFN-γ/IL-17a genes in CD44 + encephalitogenic T cells	[46]
MBD2	-	Enhanced	EAE mice	By modifying the T-bet/Hlx axis, MBD2 controls TH17 differentiation. This may be connected to the production of IFN-γ during inflammatory processes	[47]

The methylation state of certain genes, such as HLA-DRB1 and HLA-DRB5, affects risk as well as severity of MS in pathogenesis. Immune cell function is impacted and MS development is aided by hyper- or hypomethylation in important immune-related genes, such as HLA-DRB1 in CD4 + T cells and IFN-γ/IL-17a in CD44 + encephalitogenic T cells [40]. Research has also revealed modified methylation in genes governing oligodendrocyte and neuronal activities in normal-appearing white matter (NAWM), which impacts immune response and myelin integrity in multiple sclerosis. Differential methylation patterns at several genes are revealed by genome-wide methylation profiling of CD8 + T cells in MS patients, indicating a major function for controlling immune cell function, migration, and activity [37]. In MS, several genes have differential methylation patterns, such as SHP-1 and MHC2TA. Certain MS instances result in methylation alterations that quiet SHP-1, an adverse regulator of inflammation, which increases inflammation [48]. Methylated genes have been found in different parts of the brain and in neurons, which can help us understand the molecular processes underpinning brain health and illness, neurons; 31 genes with variable methylation were found in neurons after 5-methylcytosine (5mC) analysis. Important genes include RPS6KA2, MAD1L1, NOTCH4, and WHSC1, which are involved in signalling cascades and chromatin remodelling, as well as PRDM16, PRKCZ, and RPS6KA2 [49]. These results highlight the variety of molecular processes impacted by methylation modifications in neurons. Thirty-one differentially methylated genes were found in a meta-analysis of neurons; these genes overlapped with the results of the 5mC study but included distinct genes such PCSK6, ITPK1, and EIF2C2 [50]. These genes highlight the intricacy and importance of epigenetic control in neurons by being engaged in signalling cascades and protein processing. Bulk normal-appearing white matter (NAWM): 23 differentially methylated genes were found in the bulk of normal-appearing white matter (NAWM), several of which overlapped with neuronal cells [51]. These genes include WHSC1, PRDM16, and PRKCZ. SEC14L1 and RNF39 are examples of unique genes that imply pathways connected to ubiquitination and lipid metabolism, which may be disturbed in neurological diseases. Bulk hippocampus: 30 genes exhibited differential methylation in the hippocampus, an area essential for memory and learning [51]. Notable genes involved in transcription control and chromatin remodelling, which are critical for synaptic plasticity and neurogenesis, include SMYD3, TRPS1, and EIF2C2. Overlap between brain tissue and immune cells: there is evidence of a shared epigenetic environment between brain tissue and immune cells in the form of several differentially methylated genes. Genes such as WHSC1, PRDM16, and PRKCZ that overlap indicate shared pathways pertaining to immune responses and neural function [7, 52]. This overlap provides possible insights into the molecular mechanisms behind neurological illnesses by highlighting the interaction between immunological and neurological processes. Determining the molecular processes behind neurological illnesses requires an understanding of the differences in methylation patterns across various brain areas, neurons, and immune cells. These discoveries may pave the way for novel treatment approaches that focus on immune and brain cell epigenetic changes [53].

Furthermore, the found differentially methylated locations (DMPs) in T cells from MS patients and healthy controls are revealed by the analysis of DNA methylation data from these groups of people. With two sample sites and consideration of both treated and untreated patient groups, the study offers a thorough examination of the potential effects of MS and its therapies on DNA methylation patterns. Gene regulatory regions, hypomethylation in promoter regions, pathogen-related pathways in multiple sclerosis, genome-wide DNA methylation changes, treatment influence on DNA methylation, bias and confounding factors, hypermethylation in MS patient T cells, pathway enrichment analysis, and inclusion of diverse patient populations are some of the major findings [54]. They discovered substantial long-term alterations in the DNA methylation patterns of T cells from MS patients throughout the whole genome [55]. This suggests that the epigenetic landscape is dynamic and may change in response to therapeutic treatments as well as the course of the disease. Compared to untreated patients, the treated MS patient group had less significant DMPs between visits, indicating that therapy may stabilise or lessen the degree of epigenetic alterations linked to MS [20]. The difference in DNA methylation between MS patients receiving treatment and those who are not suggests a potential connection between treatment status and epigenetic changes. Patients that received therapy had lower levels of DMPs, indicating that certain medications may impact the DNA methylation pattern and perhaps stabilise the epigenome. Through the use of techniques like random placement and the inclusion of surrogate variables as covariates, the researchers considered potential biases, such as sample placement, batch effects, and biases associated with chip-based measurement [56]. The paper notes that, in spite of these adjustments, variations in DNA methylation may still be impacted by unidentified confounding variables, such as the variability of treatment regimens and sample intervals. The latest findings, which are in line with earlier research, point to a pattern of hypermethylation in CD8 + T cells from MS patients regardless of their course of therapy. This supports the theory that hypermethylation, namely in T cell-mediated immunological responses, may be involved in the pathogenesis of multiple sclerosis. In order to investigate the functional consequences of DMPs, the study conducted pathway analysis [57]. The T cell receptor signalling and death pathways were concentrated in the CD8 + T cells of treated MS patients, but the RNA transport route was enriched in the DMPs of CD8 + T cells from untreated individuals. This implies that in MS, alterations in DNA methylation may impact important cellular processes and immune system operations. Rather than in gene bodies or the 3′ untranslated regions (UTRs), the detected DMPs were primarily found in gene regulatory areas, such as the first exon [58]. This result aligns with the established function of DNA methylation in controlling the expression of genes at their promoter regions. A second research found distinct patterns of differential DNA methylation, namely demethylation linked to enhancer regions, among an underrepresented group of MS patients in the USA. The new gene-level indicators linked to multiple sclerosis and the varying reactions to therapies aimed at altering the condition. In the MS patient group, the second investigation discovered a trend of hypomethylation in putative promoter areas like TSS1500 [54]. This is consistent with research on hematologic malignancies and raises the possibility that MS and certain cancers share epigenetic pathways. Fascinatingly, the second study’s pathway analysis linked the MS patient group to bacterial and parasitic illnesses such amoebiasis and shigellosis. This is noteworthy in particular since the microbiome may play a role in the pathophysiology of MS, and these infections may be related to MS susceptibility.

The intricacy of the DNA methylation landscape in MS indicates that alterations in methylation patterns are linked to the course of the illness, the effectiveness of therapy, and maybe other hereditary or environmental variables. In order to better understand the role of DNA methylation in MS, future research should concentrate on more homogeneous patient groups, longitudinal designs with multiple time points, and larger sample sizes. These findings highlight the significance of taking epigenetic markers into account when understanding MS pathology. More research is necessary to determine whether MS, infection pathways, and epigenetic modifications linked to cancer are connected. This section highlights knowledge gaps about the methylation alterations in many genes related with MS, including T-cell factor-1 (TCF-1) and human endogenous retrovirus W (HERV-W), indicating the need for more research. All things considered, DNA methylation plays a role in MS pathogenesis by controlling gene expression, inflammation, and immune cell activity.

### Histone Modifications

**Fig. 4:**
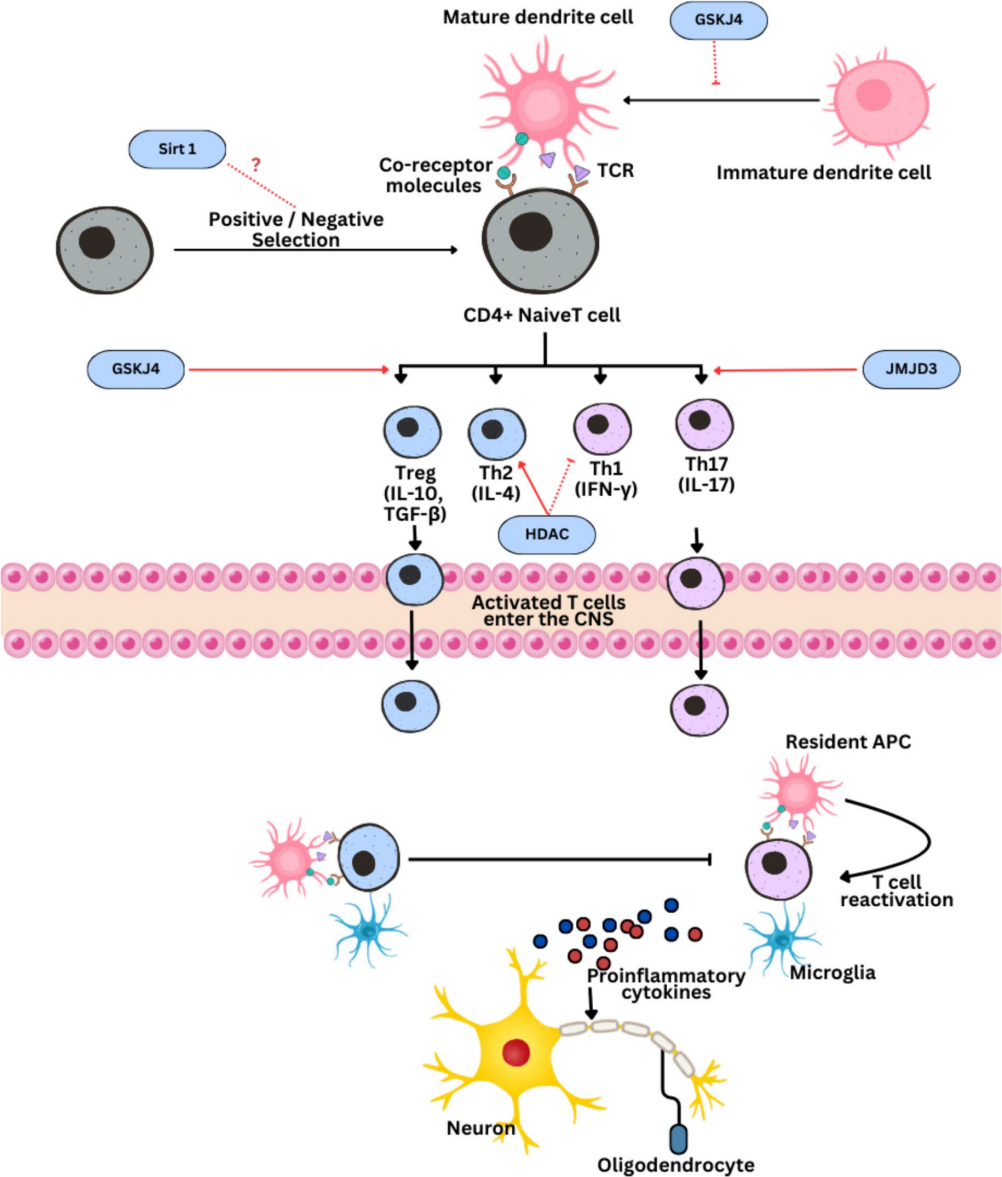
It is shown that a series of events may be responsible for MS’s autoimmunity-related demyelination and that histone-modifying enzyme inhibitors may have specific modes of action [60]

Chromatin is made up of proteins called histones, around which DNA is wrapped. The structure of chromatin can be changed by chemical alterations to histones, such as methylation, acetylation, phosphorylation, and ubiquitination. These alterations tend to relax or consolidate chromatin, increasing or decreasing the accessibility of genes to transcriptional mechanisms [59]. Through epigenetic control of gene expression, histone changes impact immunological and neurodegenerative processes, contributing significantly to the development and progression of MS. Histone changes, such as acetylation and methylation, impact gene transcription and chromatin structure in MS, changing the expression of genes associated with MS risk and the roles of the proteins they encode. Because these alterations may be used to control gene expression patterns linked to inflammation, autoimmunity, and neurodegeneration in multiple sclerosis, they provide promising targets for therapeutic interventions. Due to their effect on immune response genes, histone alterations have been connected to MS vulnerability. Histone deacetylases (HDACs), such as HDAC1, have the ability to inhibit the expression of major histocompatibility complex (MHC) genes, including HLA-DR, a crucial allele associated with multiple sclerosis. The expression of these genes may be influenced, hence lowering the risk of MS, by adjusting the levels of histone acetylation. HDAC inhibitors have shown therapeutic promise by changing the immune response from a pro-inflammatory state (Th1/Th17) to an anti-inflammatory state (Th2/Treg). This could lessen the autoimmune attack on myelin and therefore alleviate symptoms of multiple sclerosis. HDAC inhibitors block the removal of acetyl groups from histones. Histone changes in the setting of MS also control the function of CD4 + T cells, which are essential to the autoimmune pathophysiology of the illness. The development and function of several T helper cell subsets, including Th1, Th17, Th2, and regulatory T cells (Tregs), are influenced by histone acetylation and methylation. Each of these subsets plays a unique role in the advancement or suppression of illness. For instance, HDAC inhibitors can increase the growth of Tregs, which are essential for preserving immunological tolerance and lowering inflammation in MS, and block pro-inflammatory cytokines like IL-2. Furthermore, by focusing on histone methylation enzymes that alter H3K27me3 levels, one might influence the tolerogenic phenotype of dendritic cells (DCs), improving immunological tolerance and lowering autoimmune responses in MS. A model of immune mechanism in MS related to histone modification is shown in Fig. 4.

In the context of MS-related neurodegeneration and remyelination, histone changes are also crucial. The development of oligodendrocyte progenitor cells (OPCs) into adult oligodendrocytes capable of remyelination is influenced by the acetylation state of histones, namely H3 acetylation. Promoting myelin gene expression and oligodendrocyte development requires HDAC activity. Treatment strategies that alter histone acetylation may improve myelin sheath repair and remyelination in MS patients, where remyelination is frequently compromised. This might delay the course of the illness and aid in recovery. Moreover, altered histone methylation patterns, such as decreased H3K4me3, have been linked to neurodegeneration in MS, which is characterised by axonal degeneration and neuronal atrophy. These alterations may have an impact on the expression of genes related to energy metabolism and mitochondrial function, which may exacerbate atrophy and damage to neurons. Histone-modifying enzymes may be targeted in order to restore normal methylation patterns, which might provide a potential treatment approach for the neurodegenerative features of multiple sclerosis and guard against mitochondrial malfunction and neuronal death. Histone alterations in MS are generally encouraging targets for therapeutic intervention. Histone acetylation and methylation status can be used to control immune cell differentiation, lower inflammation, encourage remyelination, and fend against neurodegeneration. Histone-modifying enzyme inhibitors, such HDAC and histone methyltransferase inhibitors, may one day be turned into potent therapies for MS, addressing both the autoimmune and neurodegenerative aspects of the disease. With these therapies, MS sufferers may be able to manage their condition in a more comprehensive way and have better results.

Histone acetylation: Histones are given acetyl groups, which crack up the chromatin and increase gene accessibility for transcription. Gene expression is typically boosted as a result [61].

Histone methylation: According to the particular histone and location modified, histone methylation may produce a variety of effects. While H3K9 and H3K27 methylation are frequently linked to gene repression, histone H3 lysine 4 (H3K4) methylation is associated with active gene transcription [62].

Histone phosphorylation: Histones can acquire phosphate groups, which alters how they interact with other chromatin-associated proteins and regulates gene expression [63].

Histone ubiquitination: Heterochromatin structure and gene expression can be affected by the addition of ubiquitin molecules to histones [64].

Non-coding RNAs (ncRNAs): These RNA molecules act as regulators of gene expression rather than encoding proteins. Long non-coding RNAs (lncRNAs) and microRNAs are examples of ncRNAs that can interfere with the stability or translation of messenger RNAs (mRNAs), which can have an impact on protein synthesis [65].

Chromatin remodelling complexes: These complexes employ ATP energy to move, expel, or change the structure of nucleosomes, which affects how accessible DNA is for transcription [66].

Together, these epigenetic alterations provide an epigenetic landscape that, without altering the DNA sequence itself, can either increase or decrease gene expression. For cellular differentiation, response to environmental cues, and general organismal maturation and operation, this dynamic regulation of gene expression is essential [29]. The epigenomic molecules can be transferred from one cell to another during cell replication, marking the next generation of cells. Therefore, they can be passed on during cell meiosis and mitosis [67].

DNA methylation contributes to the diagnosis of MS, as it interferes with lymphocyte activity. DNA methylation is carried out in the brain tissues by enzymes known as DNA methyltransferases (DNMTs). DNMTs modify DNA’s cytosine bases by adding methyl groups. Because DNA methylation modifies the chromatin’s structural makeup and restricts enzyme access, it affects how genes are expressed. Specific microRNAs (miRNAs) can affect immunological and neuromodulatory processes, which can cause MS to develop when they are dysregulated [68]. Here are some typical epigenetic variables that may have an impact on the onset of MS: DNA methylation is brought on by epigenetic alterations, which promote MS progression. MS affects 20 to 30% of monozygotic twins, while it affects 5% of dizygotic twins who share the same sex [69]. Chromosome 6 displays vulnerability to MS within the genetic context. Notably, studies have demonstrated the importance of the HLA-DRB1*15:01 locus on chromosome 6 in the development of MS [70, 71]. Individuals, who are genetically susceptible to MS, do not have a strong blood–brain barrier and plaques or lesions develop in the white and grey matter. One of the main features of MS is the formation of these plaques, which result from myelin loss in the central nervous system (CNS) [4]. Each genetic locus in humans has two alleles, one of which is inherited from each parent. Therefore, the genetic inheritance of an allele in a person with MS depends on whether the gene came from the mother or the father. The importance of the major histocompatibility complex (MHC) in MS patients is highlighted by this dynamic. Notably, the HLA-DRB1*15 allele is known to be a substantial risk factor for MS, especially given that it frequently passes preferentially from mothers to their female offspring who have the disease [71]. The symptoms connected with MS can be made worse or better by epigenetic variables. The condition is known as “missing heritability” when specific genetic contributions cannot be determined by genomic mapping. This occurrence can be explained in part by the part that epigenetic pathways played in the emergence of MS [72].

###  Changes in Epigenetics in the Four Clinical Stages of Multiple Sclerosis

#### Relapsing–Remitting MS (RRMS)

Periodic relapses of neurological impairment interspersed with periods of remission are hallmarks of RRMS. During these cycles, epigenetic changes specifically, DNA methylation and histone modifications are essential for controlling immune cells. T cells in RRMS exhibit hypermethylation in genes that control immunological tolerance, including FOXP3, which lowers the activity of regulatory T cells (Tregs) and permits the dominance of pro-inflammatory Th17 cells. Relapses are made worse by this imbalance. Th17 differentiation is encouraged by dysregulated miRNAs such as miR-326, which feeds inflammatory episodes. Remission periods, on the other hand, can be associated with higher production of miR-146a, which inhibits pro-inflammatory cytokines. By altering the Th17/Treg balance in favour of immunological tolerance, therapeutic approaches that target these epigenetic regulators, such as histone deacetylase inhibitors (HDACi), may be able to extend remission [18, 73].

#### Secondary Progressive MS (SPMS)

A progression from RRMS, SPMS is characterised by decreased relapses and accumulating neurodegeneration. The shift from inflammatory to neurodegenerative processes in SPMS is reflected in epigenetic modifications: chronic axonal damage and loss of remyelination ability are caused by persistent deacetylation of histones in neurons and oligodendrocytes, which lowers the expression of neuroprotective genes. Hypomethylation of genes like TNF-α and IL-6 causes neuroinflammation and persistent microglial activation. By reestablishing the acetylation-methylation equilibrium in impacted neurons, these results highlight the significance of early epigenetic therapies to stop progression [18, 74].

#### Primary Progressive MS (PPMS)

A consistent functional decrease without noticeable relapses is a hallmark of PPMS. Oligodendrocyte progenitor cells (OPCs) in PPMS show global DNA hypomethylation, which hinders their differentiation and remyelination ability. This suggests a more stable but gradually harmful profile than that of RRMS and SPMS. By affecting oxidative stress pathways, dysregulated lncRNAs like MALAT1 lead to axonal degeneration and mitochondrial malfunction. Potential treatment targets, such as lncRNA inhibitors and substances that alter DNA methylation to improve OPC activity, are highlighted by these epigenetic disturbances [18, 75].

#### Progressive-Relapsing MS (PRMS)

Acute relapses and progressive neurological impairment are both features of PRMS. This particular combination might result from shifting epigenetic processes: StAT3-derived circ_0043813 is one example of dynamic circRNA expression that has been linked to neurodegeneration and episodic inflammation. Relapses may result from the temporary activation of pro-inflammatory genes caused by aberrant histone phosphorylation during inflammatory stages. During relapse periods, circRNAs or histone-modifying enzymes may be targeted to lessen inflammatory events and delay the course of the illness [18, 76, 77].

#### Genetic-Epigenetic Interplay at 1q21.1 Locus in Primary Progressive Multiple Sclerosis

The interaction between genetic and epigenetic factors at the 1q21.1 locus affects CHD1L expression and increases susceptibility to primary progressive multiple sclerosis (PPMS). The neurological disorder known as MS is complicated and is typified by inflammation and neurodegeneration. PPMS is a particularly difficult type of MS to treat since its underlying processes are not well understood [78]. To explore these pathways, Kakhi et. al (2024) used multi-omics data (genetic, epigenetic, and transcriptomic) from separate cohorts. The study found that PPMS patients have hypermethylation in the 1q21.1 region, which is caused by certain genetic changes and affects the expression of genes like PRKAB2 and CHD1L in brain tissues. A clear connection between DNA methylation and gene expression was shown by functional investigations utilising reporter assays and CRISPR/dCas9 [78]. This association was further corroborated by correlation network analysis, which linked these genes to PPMS brain functions. CHD1L plays a crucial role in maintaining the integrity of neurons since its downregulation or deletion causes developmental and functional abnormalities in both zebrafish models and human iPSC-derived neurons. According to the research, 1q21.1 region DNA methylation and gene expression are impacted by genetic variations linked to PPMS. This information may be important for comprehending the pathophysiology of PPMS and may also serve as a target for future treatment approaches.

#### Non-coding RNAs (ncRNA) like circRNAs, lncRNAs, miRNAs, and MALAT1 in MS

It is difficult to create successful MS medicines since there are no particular diagnostic or prognostic indicators. However, because of their functions in a variety of biological processes including immunology and inflammation, ncRNAs such as circRNAs, lncRNAs, miRNAs, and metastasis-associated lung adenocarcinoma transcript 1 (MALAT1) have lately attracted interest as promising molecular tools for early diagnosis and therapy of MS [65]. A major factor in the activation, differentiation, and activity of T and B cells, as well as a contributing factor to the disease’s development, is the dysregulation of miRNAs in MS, which is intimately linked to CNS damage.

Certain miRNAs, including miR-155, a well-known biomarker of MS development, miR-155 is essential for inflammatory reactions and immune cell activation. When it is dysregulated, more pro-inflammatory cytokines are produced, which encourages the activation of T cells and monocytes. Since active MS lesions and the advancement of the illness have been repeatedly associated with elevated miR-155 levels, this protein may be a target for therapeutic intervention to alter immune responses [79]. Relapsing–remitting MS (RRMS) patients have Th17 cells that are overexpressed with miRNAs such as miR-326, which block Ets-1 and so enhance Th17 cell development. In MS patients, other miRNAs including miR-590 and miR-27a are also increased and support Th17 cell development via several molecular pathways like TGF-β and Tob1 signalling [80]. It has been demonstrated that increased levels of miR-448 and let-7e raise Th1 and Th17 cell ratios, respectively, and trigger a Th17 response through the PTPN2 pathway. Apart from T cells, miRNA dysregulation in B cells, such as miR-326 and miR-155, promotes B cell development into CNS-targeting antibody-producing plasma cells. This is made worse by the breakdown of the BBB brought on by downregulated miR-320a and increased MMP-9. Moreover, in the B cells with RRMS patients, dysregulated miRNAs such the miR-106b-25 and miR-17–92 clusters impact pathways of signalling including the B cell receptor and PI3K, which increases the production of cytokines, including TNF-α because of miR-132 overexpression [81]. Additionally, miRNAs such as miR-146a and miR-155, which affect dendritic cell activation and encourage macrophage M1 polarisation, respectively, play critical roles in the pathogenesis of multiple sclerosis. However, the differentiation of macrophages into the M2 phenotype, which has anti-inflammatory potential in MS patients, has been associated to miR-124 downregulation [82].

The potential of MALAT1, a crucial lncRNA in multiple sclerosis, as a therapeutic target to restore normal splicing patterns is highlighted by its association with dysregulated splicing factors and aberrant alternative splicing that impact important pathogenic genes. Additionally, by modifying glutamate receptor signalling and lowering excitotoxicity, ncRNAs like as miR-223-3p and miR-27a-3p demonstrate neuroprotective benefits. Because of their functions in controlling immunological responses, neurodegeneration, and epigenetic changes, ncRNAs like miR-155 and MALAT1 together function as indicators for the advancement of illness and potential therapeutic targets. To fully understand their therapeutic potential, more study is essential.

Increasing evidence supports the involvement of lncRNAs in MS pathogenesis. LncRNAs, which are more than 200 nucleotides long, have been implicated in various functions, including chromatin remodeling, miRNA sponging, and protein stability regulation. These molecules contribute to numerous neuronal biological processes such as CNS development, myelination, neurotransmission, and neuroinflammation [83]. Dysregulated lncRNAs, like growth arrest-specific transcript 5 (GAS5) and MALAT1, are implicated in MS. GAS5 upregulation suppresses TRF4 transcription, inhibiting M2 polarisation, while MALAT1 downregulation promotes macrophage polarisation towards an M1 inflammatory phenotype and alters Th1 and Th17 cells, reducing T-reg differentiation. Other lncRNAs like PANDA, TUG1, and NEAT1 are associated with immune responses and DNA damage in MS patients. PANDA, involved in p53-mediated DNA damage response, leads to apoptosis and free radical production when dysregulated. TUG1, upregulated in RRMS patients, targets miR-20a-5p and activates p38 MAPK, promoting inflammatory cytokine production. NEAT1 increases IL-8 levels via SFPQ modulation, contributing to inflammation [84]. Additionally, lncRNAs like UCA1 and linc-MAF-4 regulate T cell proliferation and Th1/Th2 ratios, respectively, enhancing pro-inflammatory processes. LncDDIT4, upregulated in MS, modulates immune response through Th17 differentiation by influencing the mTORC1 pathway and IL-17 production [85].

One important feature of MS is disruption of the BBB, which is important for the disease’s progression. The integrity of the blood–brain barrier is compromised as a result of the disruption of adherens junctions (AJs) and tight junctions (TJs) between endothelial cells. Neurodegeneration results from such injury, which makes it possible for immune cells and other dangerous chemicals to enter the brain. The BBB disruption seen in MS is also influenced by cytoskeletal protein alterations. circRNAs have been implicated in maintaining BBB integrity and regulating the cytoskeleton, according to recent research [86]. CircRNAs are special non-coding RNAs that control the expression of genes and give proteins structural support. Numerous circRNAs have been discovered to affect important proteins that are part of the cytoskeletal architecture of the BBB, including alpha-actinin1 (ACTN1) and TJ proteins such occludin, claudin-5, and ZO-1. For example, hsa_circ_ACTN1_2453 suppresses ACTN1 expression, but hsa_circ_DLGAP4 buffers miR-143, which suppresses BBB integrity via controlling TJ proteins. One potential treatment strategy for multiple sclerosis is to target circRNAs. CircRNAs are becoming more and more popular as prospective biomarkers and therapeutic targets because of their closed circular form and tissue selectivity [87]. CircRNA expression changes in MS patients’ blood leukocytes, such as the downregulation of circ_0035560 and the overexpression of circ_0005402 and hsa_circ_0106803, indicate their role in the disease’s pathophysiology. GSDMB’s relevance in MS is further validated by its function in cytokine production and its effect on TGF-β1 and MMP9 expression levels. GSDMD activation is a possible biomarker for MS since it has been connected to inflammasome-signalling-dependent pyroptosis, which increases inflammation in the illness [88]. In MS models, circ_HECW2, another circRNA, is linked to brain development regulation and BBB failure. Its effects on apoptosis and cell proliferation, as well as its control of NEGR1 expression through miR-30e-5p sponging, point to its regulatory involvement in MS pathogenesis [80]. Similarly, Th17 differentiation and inflammatory processes associated with multiple sclerosis are impacted by dysregulation of circ_0043813, which is generated from the STAT3 gene. Sophisticated methods like high-throughput sequencing and spatial transcriptomics can help find circRNAs that control BBB function. Creating circRNA-based treatments may enhance patient outcomes, stop the spread of illness, and preserve the integrity of the blood–brain barrier.

Due to their participation in genetic control and disease-related signalling pathways, lncRNAs play a key role in the course of MS. The intricacy and significance of lncRNA networks in MS have been demonstrated by bioinformatics analysis. Han and colleagues, for instance, discovered that 517 of the 2,383 brain-specific lncRNAs that were differently expressed in 142 people were impacted by SNPs. SNPs have a major influence on lncRNA expression, namely on antigen processing/presentation and MAPK pathways, which are important in autoimmune inflammatory disorders, according to their eQTL research [89]. According to Sabaie et al., the MAPK pathway is important for the pathophysiology of MS, and bioinformatics has shown lncRNA-associated ceRNA regulation networks in the periplaque areas of MS patients. Three important lncRNAs (TUG1, ASB16-AS1, LINC01094), nine miRNAs, and five hub genes were shown to be part of a network that is significantly involved in MAPK signalling and other pathways. In the investigation of MAPK14-related lncRNAs, Ghafouri-Fard et al. discovered that MS patients had upregulated levels of NORAD and RAD51-AS1, suggesting dysregulation in MAPK14-related lncRNAs; however, they did not correlate with the severity of the condition [90, 91]. Three key lncRNAs (XIST, OIP5-AS1, and CTB-89H12.4) were found by Ding et al. in an MS-related lncRNA-ceRNA network, and their roles in RNA transport and mTOR signalling were revealed [92]. NF-κB-associated lncRNAs were studied by Safa et al., they discovered that MS patients have changed amounts of these lncRNAs’ expression, which may have an impact on the disease’s processes [93]. In EAE models, Zhang et al. concentrated on lncRNA AK018453 and the TRAP1/Smad pathway. They found that TRAP1/Smad pathway activation is connected to the production of pro-inflammatory cytokines, suggesting it as a possible therapeutic target [94]. Liu and colleagues brought attention to the function of lncRNA Gm13568 in controlling the Notch1 pathway, demonstrating its relevance in demyelination and inflammation. Through its interaction with miR-686, Bian et al. investigated the effect of lncRNA Gm15575 on Th17 cell development and discovered that it is essential in MS aetiology [95]. Key lncRNAs (LINC00649, TP73-AS1, MALAT1) and possible therapeutic medicines were found by an integrated study by Wang et al., offering insights into MS processes and prospective treatment approaches [96]. All in all, these investigations highlight how important lncRNAs are in controlling important signalling pathways in multiple sclerosis, providing prospective targets for upcoming treatment approaches. According to research on lncRNAs in MS, these molecules have a critical role in controlling immune responses and important inflammatory pathways. LncRNAs that modulate disease processes, such as TUG1, Gm13568, and AK018453, may be useful as biomarkers and targets for therapeutic intervention in multiple sclerosis [80]. Overall, research on ncRNAs, such as circRNAs, lncRNAs, and miRNAs, offers important new understandings into the pathophysiology of MS. Their participation in immunological and inflammatory responses, as well as their expression levels and processes, present intriguing directions for the development of new diagnostic and prognostic tools for the illness [80].

The relationship between epigenetic modifications and immunological response/autoimmunity in MS highlights the complex regulatory processes that affect the onset and progression of this difficult neurological condition. To identify the underlying molecular mechanisms causing MS pathogenesis and to create individualised and targeted therapy strategies, a thorough understanding of these epigenetic changes is essential. MS aetiology has been linked to epigenetic disruption. Numerous studies have demonstrated the importance of epigenetic changes in the onset of MS, with many epigenetic targets playing a key role [97]. Table 2 highlights the complexity of epigenetic dysregulation in MS by summarising its effects and underlying mechanisms. In summary, the enormous influence that epigenetic changes have on gene expression without changing the DNA sequence suggests a viable direction for the creation of cutting-edge therapeutics for MS.

**Table 2:** Comprehensive details on the many epigenetic targets and the various mechanisms like DNA methylation, histone modifications, microRNAs, and regulatory factors contribute to immune dysregulation and neurodegeneration linked to the pathophysiology of MS. Important discoveries include the discovery of new biomarkers, the part that particular epigenetic pathways play in the development of the illness, and their potential as MS treatment targets

Type of study	Study outcome	Mechanism of epigenetic dysregulation	Reference
Comprehensive literature review	This study revealed the potential of trained immunity as a therapeutic target while examining its involvement in autoimmune disorders. The study especially focuses on multiple sclerosis, rheumatoid arthritis, systemic lupus erythematosus, and type 1 diabetes to emphasise the impact of epigenetic and metabolic changes in innate immune cells going through a training process	The mechanism involves epigenetic and metabolic alterations in innate immune cells that are linked to trained immunity, which may have an impact on the aetiology of autoimmune disorders by altering the balance between helpful and detrimental immune responses	[98]
Research: an observational study	In this study of circRNA expression in MS patients, dysregulated circRNAs among them hsa_circ_0007990 were found that may serve as RNA-based biomarkers. The study suggests that circRNA expression and genetic variations associated with MS have a role in disease aetiology. A link between exon methylation and circRNA expression levels is also suggested by the study, pointing to the role of epigenetic mechanisms in circRNA control	According to the findings, exon-based DNA methylation may be a crucial factor in controlling the expression of circRNA, thus contributing to the dysregulation seen in MS patients	[99]
Comprehensive literature review	The function of B lymphocytes in autoimmune disorders and the impact of translational factors and epigenetic controls on their development were revealed by this study. Multiple autoimmune diseases such as multiple sclerosis, lupus, arthritis, diabetes, Sjögren’s syndrome, and pemphigus, are influenced by the dysregulation of these variables. A literature review is part of the study, which explains how epigenetic dysregulation contributes to the autoimmune B cell response	B cell development and differentiation are impacted by altered transcriptional factors and epigenetic alterations, which play a role in the aetiology of autoimmune diseases like MS	[100]
Comprehensive literature review	This review emphasises the complex nature of remyelination in MS and shows the limits of remyelination therapies that only target particular cell types or pathways. Remyelination failure is primarily caused by dysregulated processes in different cell types and their impaired coordination. The study uses a literature review method to summarise the available data, highlighting the need for a thorough comprehension of the complicated cell interactions at CNS lesion locations for successful medication development in MS	Remyelination failure is primarily caused by dysregulated processes in different cell types and their impaired coordination	[101]
Comprehensive literature review	The review emphasises the significance of the miR-183/96/182 cluster (miR-183C) in immune cell activity and how it is dysregulated in MS and other autoimmune diseases such as systemic lupus erythematosus (SLE) and ocular autoimmune diseases. MiR-183C is emphasised for its effect on immune cell development and function as it is discussed in its potential application as a biomarker and therapeutic target in MS	The epigenetic dysregulation mediated by miR-183C contributes to immune dysfunction and the pathogenesis of MS	[102]
Clinical research article	Rheumatoid arthritis, psoriasis, and multiple sclerosis have different immune response patterns, according to the study, which looks at SARS-CoV-2 infection in patients with pre-existing autoimmune diseases. Peripheral blood mononuclear cell single-cell study revealed variations in gene expression, transcription factor activity, and cell–cell communication. Notably, changes in type-I interferon signalling and inflammatory T cell and monocyte responses were seen, highlighting the necessity of patient monitoring and individualised treatment plans	Variations in gene expression, transcription factor activity, and cell–cell communication	[103]
Experimental study involving in vitro and in vivo modeling	This study revealed the significance of the protective histone deacetylase 7 variation (HDAC7.p.R166H) in MS. It shows that the variation increases the suppressive ability of Foxp3 + regulatory T cells (Tregs), which may have an impact on the severity of the disease. Additionally, a knock-in mouse model is used in the work to imitate the human HDAC7 R166H variation and shed light on the accompanying transcriptome changes. These results emphasise the potential influence of epigenetic modifiers on the beginning of MS	Protective HDAC7 variation boosts Tregs’ ability to inhibit MS, potentially impacting its severity	[104]
A case–control study comparing	In this investigation, the expression levels of miR-146a and miR-155 in the serum of MS patients and healthy controls are examined. The expression of these microRNAs was found to be dysregulated, notably in vitamin D levels, disease impairment, and the frequency of attacks in MS patients. The study emphasises the potential involvement of miR-146a and miR-155 in the development and progression of MS	The dysregulation of miR-146a and miR-155 expressions, which may be altered by epigenetic factors, and their correlation with vitamin D insufficiency, disease impairment, and the frequency of attacks in MS patients are the mechanisms at play	[105]
Comprehensive literature review	It emphasises how mutations, epigenetic changes, and variations in the expression of mitochondrial genes cause inflammation and neurodegeneration. Providing insights into the mechanisms of epigenetic dysregulation associated with mitochondrial dysfunction in the context of neurodegenerative disorders such as Alzheimer’s, Parkinson’s, multiple sclerosis, and amyotrophic lateral sclerosis, approach to emphasise the potential of mitochondria as a drug target for the regulation of neurodegenerative diseases	Mutations, epigenetic changes, and variations in the expression of mitochondrial genes cause inflammation and neurodegeneration	[106]
In vitro, ex vivo and in vivo models	Through the discovery of differentially expressed and methylated genes in dermal fibroblasts (dFBs), the study sheds light on the function of epigenetic dysregulation in the development of systemic sclerosis (SSc). Important transcription factors and microRNAs, including KLF4, TBX5, TFAP2A, miR-10a, and miR-10b, were discovered to be implicated, with KLF4 showing antifibrotic properties. The possibility of using epigenetic modifiers as therapeutic therapies in SSc is highlighted by these findings. There are worries that multiple sclerosis may develop through comparable epigenetic dysregulation pathways	Epigenetic dysregulation in transcription factors and microRNAs, including as KLF4, TBX5, TFAP2A, miR-10a, and miR-10b	[107]
Combining population genetic studies	To better understand the involvement of Epstein Barr virus (EBV) in the pathogenesis of multiple sclerosis (MS), the study suggests an integrated approach that combines population genetic studies, immunology, and molecular virology. The research tries to reconcile the seemingly disparate ideas on the genetic and viral origins of MS by reevaluating the link between genetic and environmental factors. By illuminating the intricate function that EBV plays in the disease, this strategy may open the door to developing methods to prevent MS	Genetic and immunological variations due to ESV	[108]
Comprehensive literature review	This study revealed that the development of multiple sclerosis (MS) is significantly influenced by microglia and microRNAs (miRNAs). It emphasises how dysregulated microRNAs affect the polarisation and activity of microglia, contributing to MS inflammation and neurodegeneration. Specific microRNAs are miR-155, miR-145, miR-125b, miR-222, miR-32, miR-142, miR-223, miR-124, miR-146a, miR-204, miR-17, miR-199β, and miR-7. The major goal of the project is to identify specific miRNAs that could be exploited as MS therapy targets	In the context of MS, the mechanism involves the dysregulation of certain miRNAs that impact microglial polarisation, affecting inflammatory reactions and myelination	[109]
Systematic literature review	This study revealed miRNA dysregulation in MS and emphasised the impact of particular miRNAs on CD4 + cell differentiation and the balance of inflammatory response, such as miR-141, miR-200a, miR-155, miR-223, and miR-326. It implies that their dysregulation is a result of deviations in the stages of miRNA synthesis, which may be impacted by epigenetic changes	The mechanism involves the impact of epigenetic changes on the transcription of the miRNA gene, which can affect the expression and function of particular miRNAs implicated in the control of CD4 + cell differentiation and the balance of inflammatory responses in MS	[110]
Review	The review places a strong emphasis on the function of monocytes in MS and other neurodegenerative illnesses like Parkinson’s disease, Alzheimer’s disease, and amyotrophic lateral sclerosis. It emphasises the role of monocytes in CNS inflammation and their influence on the course of disease. The information offered sheds light on the various roles played by monocyte subsets, their cytokine release, and phenotypic changes in the context of multiple sclerosis	Monocytes in CNS inflammation and their influence on the course of disease	[111]
A combination of bioinformatics analysis	The epigenetic processes regulating the equilibrium of T helper (Th)1 and Th2 cells as well as autoimmune reactions in MS. The study discovered a competitive endogenous RNA network made up of six long noncoding RNAs (lncRNAs), 21 miRNAs, and 86 mRNAs by using bioinformatics analysis and molecular assessments. Through the use of quantitative real-time PCR and the Gene Expression Omnibus, it was shown that several important genes, including NEAT1, MALAT1, RUNX3, GATA3, and TBX21, as well as miR-544a and miR-210-3p, were dysregulated. The study introduced potential novel biomarkers linked to the Th1/Th2 imbalance in MS by confirming an imbalance in IFN- (Th1)/IL-4 (Th2) levels in MS patients	lncRNAs, miRNAs, and key transcription factors as novel biomarkers for the Th1/Th2 imbalance in MS	[112]
Research: a comprehensive analysis	This study emphasises CD4 + and CD19 + cells’ major roles in the pathogenesis of multiple sclerosis (MS) by highlighting the altered chromatin-accessibility profiles in immune cells of MS patients compared to healthy controls	Chromatin areas with genetic variations linked to MS, differentially methylated locations, and dysregulated gene expression in CD4 + cells	[113]
Comprehensive literature review	The research reveals that the epigenetic dysregulation seen in MS is significantly influenced by RNA produced from retroelements. Retroelement activity contributes to the complex pathogenesis of MS and other neurological disorders like Aicardi-Goutieres syndrome (AGS), Rett syndrome (RTT), amyotrophic lateral sclerosis (ALS), Alzheimer’s disease (AD), schizophrenia (SZ), and ageing through mechanisms like chromatin remodelling, altered splicing, and modulation of transcript abundance. It is essential to comprehend these epigenetic pathways to identify prospective treatment targets and create plans for dealing with the complexity of MS progression	The epigenetic dysregulation seen in MS is significantly influenced by RNA produced from retroelements	[114]
Comprehensive literature review	The focus of this study is on how lifestyle choices affect miRNA expression dysregulation and its effects on a range of biological processes. It emphasises the function of miRNAs in cell cycle control, carcinogenesis, and inflammation, all of which may have a role in the emergence of illnesses. The work emphasises the need to examine these connections for new therapeutic approaches and disease prevention, particularly in light of the development of multiple sclerosis (MS)	miRNA expression dysregulation	[115]
Comprehensive literature review	This study states that the alterations in gene expression through DNA methylation, histone modification, and microRNA-mediated gene regulation contribute to dysregulated immune responses and neurodegenerative processes in MS	DNA methylation, histone modification, and microRNA-mediated	[116]
Extensive literature review	Glucocorticoids, oestrogens, prolactin, dehydroepiandrosterone, melatonin, and vitamin D are among the key hormonal influences on MS that are highlighted in this thorough analysis. They are emphasised in terms of the pathophysiology of MS, the severity of the condition, the relationship to mental illnesses, and fertility. About disease-modifying therapies and the condition of the hypothalamic–pituitary–adrenal (HPA) axis, the study emphasises the importance of the pre-and post-pregnancy phases in MS patients	The interaction of hormonal elements and epigenetic regulation of immunological dysregulation, psychological factors, cognitive status, and fertility in MS patients are all part of the system. The review emphasises the importance of conducting more studies in this area	[117]
Systematic literature review	The relationship between smoking, genetics, and environmental variables and multiple sclerosis (MS) is the main emphasis of this study. It emphasises the important role that smoking plays as a risk factor for MS and the need to modify environmental and lifestyle factors in disease prevention	The mechanism involves the interaction between smoking and epigenetic changes, which may aid in the development of immunological dysregulation in MS	[118]
Experimental study in animal models	The cuprizone mouse model of MS used in this study shows the advantages of betaine supplementation. Betaine efficiently restores epigenetic control, enhances neurological function, and lessens axonal damage by raising H3K4me3 levels and the SAM/SAH ratio. Betaine also enhances neuronal respiration and activates transcriptional programmes that are neuroprotective, reducing the axonal damage linked to mitochondrial dysfunction in MS and its symptoms	The mechanism involves the restoration of epigenetic control and activation of neuroprotective transcriptional programs by betaine, likely through modulation of the SAM/SAH ratio and histone methyltransferase activity, particularly H3K4me3, which supports neuronal energetics and mitigates axonal damage in MS	[119]
Cross-sectional study	The purpose of the study was to compare the expression of miR-326 in individuals with relapsing–remitting multiple sclerosis (RRMS) who responded and did not respond to interferon-beta (IFN-) therapy. The results indicate the involvement of different epigenetic processes in the regulation of Th17 development because IFN treatment does not appear to significantly alter the expression of miR-326	The findings of this work imply that the normalisation of miR-326 expression may not be the only epigenetic method by which IFN-β may influence Th17 development, pointing to the potential involvement of additional epigenetic mechanisms	[120]
Comprehensive literature review	The review puts up the idea that the disruption of nervonic acid supply caused by decreased SCD from vitamin D deficiency may have an impact on myelin instability and the imbalance between harmful CD4 + Th17 cells and beneficial CD4 + Treg cells in multiple sclerosis (MS). It implies that 1,25-dihydroxyvitamin D3 signalling enhances epigenetic tagging and reinstates the dominance of Treg cells over Th17 cells. The study emphasises the effect of vitamin D deficiency on epigenetic dysregulation in MS by integrating investigations of myelin stability and immune cell balance	The imbalance between CD4 + Th17 cells and CD4 + Treg cells in multiple sclerosis and myelin instability may be impacted by the decrease in SCD caused by vitamin D insufficiency	[121]
Comprehensive literature review	The review emphasises the function of the Regulator of G protein signalling 10 (RGS10) in controlling physiological processes in many cells as well as its relevance to several disorders, such as Parkinson’s disease, multiple sclerosis, osteopetrosis, chemoresistant ovarian cancer, and cardiac hypertrophy. It offers insights into RGS10’s regulatory processes, focusing on how epigenetic mechanisms and post-translational changes affect the expression, location, and function of RGS10 by controlling its transcription	RGS10’s characteristics and roles in cellular physiology and disease aetiology	[122]
Comprehensive literature review	The study demonstrates a novel method for identifying certain cell types that are impacted by risk loci for MS. It reveals risk loci exclusive to myeloid and the central nervous system, including those that alter oligodendrocytes’ escape from transcriptional pauses. This process’ dysregulation prevents oligodendrocytes from maturing, which prevents myelin renewal and causes neuronal loss. These results point to the possibility of novel treatment targets for MS pathogenesis, including cell-intrinsic abnormalities beyond the immune system	Dysregulation prevents oligodendrocytes from maturing, which prevents myelin renewal and causes neuronal loss	[123]
Research: an observational study	In the post-mortem brain tissue of MS patients, this study discovered significant DNA methylation and hydroxymethylation alterations in neuronal nuclei. Reduced transcriptional activity was linked to these alterations, particularly those within gene bodies. Axonal guidance and synaptic plasticity-related genes were shown to be dysregulated, with a substantial effect on CREB signalling. Reduced CREB activity was confirmed by immunohistofluorescence examination in the white matter neurons of MS patients, suggesting a potential contribution to the compromise of neuro-axonal integrity in MS	The study reveals persistent hypo-5mC and hyper-5hmC changes in MS patients’ neurons, suggesting that these changes may compromise CREB-mediated neuro-axonal integrity and contribute to the clinical symptoms of MS	[124]
Case–control study	Downregulation of GSTT1-AS1 and IFNG-AS1 lncRNAs was found in this study of Iranian MS patients, suggesting their participation in immune response dysregulation. Despite increased expression of TNF and IFNG, the alterations were not statistically significant. SYBR Green-based Real-time quantitative PCR was used in the study to evaluate the levels of lncRNA and gene expression in peripheral blood samples. These findings shed light on the potential contribution of epigenetic pathways, particularly through dysregulated lncRNA networks, to the onset of MS	Downregulation of GSTT1-AS1 and IFNG-AS1 lncRNAs	[125]
Integrated literature review	The study emphasises the intricate interplay between genetic predispositions, environmental exposures, and epigenetic changes that contribute to the start of MS, which is multifactorial. It shows the significance of gene-environment (GxE) interactions in immune homeostasis dysregulation and the necessity for additional studies to uncover prognostic indicators, particularly during the neurodegenerative stage of the disease	Genetic changes that contribute to the start of multiple sclerosis	[21]
Meta-analysis	A potential role in MS pathogenesis through the dysregulation of alternative splicing of MS-associated genes is suggested by MALAT1 overexpression in MS. The study validated MALAT1 overexpression in MS cohorts by using a meta-analysis of microarray data. Experiments with MALAT1 manipulation showed its effect on splicing factors and AS of key genes, highlighting its participation in aberrant splicing profiles associated with MS	The dysregulation of alternative splicing of MS-associated genes is suggested by MALAT1 overexpression in MS	[126]
GWAS analysis	This study reveals that miRNAs influence immunological signalling and other pathways, contributing to the aetiology of paediatric MS (ped-MS). Candidate miRNA-target gene pairs connected to immunological and neural signalling were discovered by enrichment analysis. MiR-SNP study connected immunological signalling pathways to dysregulated miRNA binding. The relevance of miRNAs in ped-MS and their potential for diagnostic and therapeutic use are revealed by the findings	MiR-SNP study connected immunological signalling pathways to dysregulated miRNA binding	[127]
Research: experimental models	According to the study, the upregulation of miR-223-3p and miR-27a-3p during inflammation has the protective effect of reducing neurons’ sensitivity to glutamate through modifying glutamate receptor signalling. This emphasises the potential of these microRNAs as therapeutic targets for controlling neurodegeneration brought on by multiple sclerosis	The upregulation of miR-223-3p and miR-27a-3p	[128]
Comprehensive literature review	The study revealed the significance of non-coding RNAs (ncRNAs) as important epigenetic regulators in multiple sclerosis (MS), emphasising their role in the development of the disease. There is a significant potential for ncRNAs, particularly long (I) ncRNAs and micro (mi) RNAs, in the diagnosis and a potential target in the therapy of MS	The mechanism involves the regulatory functions of ncRNAs, particularly long (l) ncRNAs and micro (mi) RNAs, in altering gene expression patterns, contributing to the pathogenesis of MS through epigenetic modifications	[129]
Research	In both cultured human astrocytes and MS lesions, the study shows that the genetic risk mutation rs7665090G near NFKB1 impacts astrocytes, increasing NF-B signalling and enhancing lymphocyte migration. Due to this imbalance, autoimmune inflammation in MS is facilitated by peripheral immune cells' access to the CNS	The genetic risk mutation rs7665090G near NFKB1 impacts astrocytes, increasing NF-B signalling and enhancing lymphocyte migration	[130]

## Mitochondrial Dysfunction in Multiple Sclerosis

### Overview of Mitochondria as Cellular Organelles Responsible for Energy Production and Nerve Cell Function

All eukaryotic cells contain mitochondria, which are primarily responsible for supplying the cells with energy in the form of adenosine triphosphate (ATP) through the oxidation of metabolic fuels. They also participate in numerous other critical physiological processes, such as calcium homeostasis, apoptosis, and fatty acid oxidation. Mitochondria are made up of a matrix, an inner and outer mitochondrial membrane (IMM and OMM). The IMM is the site of the mitochondrial electron transport chain (ETC), which is made up of five protein complexes: Complex I (NADH dehydrogenase), complex II (succinate dehydrogenase), complex III (ubiquinone: cytochrome c oxidoreductase), complex IV (cytochrome c oxidase), and complex V [adenosine triphosphate, (ATP) synthase]. The Krebs cycle, also known as the tricarboxylic acid (TCA) cycle, occurs in the mitochondrial matrix and produces nicotinic adenine dinucleotide (NAD)H and flavin adenine dinucleotide (FAD)H2, which are then utilised as electron donors in the ETC [131, 132]. The ETC moves protons out of the mitochondrial matrix and into the intermembrane gap. This generates an electrochemical proton gradient across the IMM, sustaining the mitochondrial membrane potential (MMP) while also producing ATP. It is crucial to maintain the MMP. While a high MMP increases ATP synthesis and superoxide generation, a low MMP is linked to limited ATP, low superoxide production, and mitochondrial removal through autophagy [131, 133]. Mitochondria are extremely dynamic organelles that are essential for the production of ATP. Mitochondria perform several essential cellular processes in addition to making ATP, including controlling oxidative stress, maintaining the balance of intracellular Ca^2+^ signalling, and synthesising steroids. Ion homeostasis, cell signalling, and death are all regulated by Ca^2+^ accumulation in mitochondria. These are all essential for CNS cells, particularly for neurons because of their quick metabolism and synthesising higher susceptibility to oxidative damage. Additionally, ATP is necessary for neurons to function properly in terms of neurotransmission and plasticity. As a result, mitochondrial function is crucial for the healthy development of neuronal and survival [131]. Reactive oxygen species (ROS) production and oxidative Brandria [134, 135]. As a consequence of oxidative metabolism, mitochondria produce ROS when molecular oxygen undergoes a one-electron reduction to form a superoxide anion [136]. Due to their high energy requirements and vulnerability to energy deprivation, healthy oligodendrocytes (OLs) are likely dependent on the presence of functional mitochondria [137]. Numerous studies have shown that NO interferes with mitochondrial function, which in turn impacts how oligodendrocytes use energy. Overproduction of ROS by dysfunctional mitochondria can cause the peroxidation of lipids, proteins, and DNA. This can cause axonal energy failure, which can then cause neurodegeneration and the activation of apoptotic pathways [135, 138].

Reactive oxygen species (ROS), which are important for ATP generation and calcium control, are primarily produced by mitochondria, an important intracellular organelle. Diffuse mitochondrial failure brought on by MS disease processes may therefore result in insufficient energy generation and intracellular dysregulation. Given their distinctive elongated architecture and reliance on ATP to transmit electrical signals, maintain ionic gradients, and promote anterograde and retrograde movement through axons, neurons are particularly vulnerable to the effects of this dysfunction. Eukaryotic cells only contain circular, non-nuclear DNA in their mitochondria, leaving a trace of their bacterial ancestry. The complex intramitochondrial machinery that drives oxidative metabolism, the oxidative phosphorylation chain (OxPhos), is made up of 13 key proteins and 22 transfer RNAs that are all encoded by mitochondrial DNA (mtDNA). Additionally, reactive oxygen species (ROS), which are crucial for signalling, are continuously produced by mitochondria. However, oxidative stress and significant damage to vital cell components can result when the rate of ROS production surpasses the cellular antioxidant capability [139].

Due to alterations in energy demand and energy production brought on by the disease, many of the normal mitochondrial activities appear to have been disrupted in MS-related neurons and OLs from postmortem tissue and animal models of the disease [140]. There is no definitive triggering event in MS that causes mitochondrial abnormalities. Studies using immunohistochemistry (IHC) on post-mortem brain tissue and measurements of the oxygen consumption rate (OCR) in human cell lines lead to the conclusion that neuronal mitochondrial dysfunction in the MS cortex may be a key factor in the disease’s pathology and may be related to poor myelination and axonal degradation [141].

### Mitochondrial Dysfunction in the Pathogenesis of Multiple Sclerosis: Including Role of Oxidative Stress and Mitochondrial Abnormalities

The most frequent cause of neurological disability in young people is MS, an autoimmune-mediated condition that affects the CNS. Areas of demyelination that occur in the cerebral white and gray matter are the predominant symptom of this illness. The two main pathogenic characteristics of MS are inflammation, which is followed by demyelination, and astroglial growth (gliosis), which is accompanied by neurodegeneration. Primary progressive multiple sclerosis (PPMS) and relapsing–remitting multiple sclerosis (RRMS) are the two main types of MS that have been identified based on clinical traits, disease history, and aetiology [142].

Demyelination, inflammation, and neurodegeneration in the central nervous system are symptoms of MS. In the aetiology of MS, mitochondrial dysfunction plays a key role in the pathophysiology and development of the illness [143]. Mitochondrial dysfunction’s function oxidative stress, inflammation, and poor energy metabolism in MS are caused by disrupted mitochondrial dynamics, reduced ATP synthesis, and increased generation of reactive oxygen species (ROS) [144]. MS patients and animal models both have mitochondrial abnormalities, which exacerbate demyelination and neuronal damage. These abnormalities include mutations, poor DNA repair, and altered enzyme function [145]. Axonal demyelination, excitotoxicity, and finally neuronal apoptosis are caused by mitochondrial malfunction, which also causes an imbalance in the neurotrophic support system, a reduction in N-acetylaspartate synthesis, and energy deprivation in neurons and oligodendrocytes [146]. Relationship between epigenetics and autoimmune disease because of the increased oxidative stress and reduced energy production caused by mitochondrial dysfunction in MS, autoreactive lymphocytes, and chronic neuroinflammation is activated [146, 147].

The disruption of mitochondrial activity may result from epigenetic alterations driven by environmental variables, which could exacerbate the autoimmune response and accelerate the course of MS [148]. The intrinsic process of apoptosis, which causes cancerous or damaged cells to die, directly involves mitochondria. Several cell death events trigger the mitochondrial pathway, including DNA damage, exposure to chemotherapy medicines, UV rays, and serum depletion. Apart from being the spot of interaction between pro- and anti-apoptotic proteins, the mitochondrion is a origin of transmitted signals that cause caspases, the primary apoptotic enzymes, to become activated. As a result, several mitochondrial proteins that ordinarily perform different roles initiate the function of the caspases during apoptosis. In neurodegenerative diseases like MS, mitochondrial-dependent apoptosis can directly cause neuronal death [149, 150].

Inflammation is a pathological response that is seen in the body from the moment autoreactive lymphocyte forms first appear in peripheral tissues throughout the entire course of MS development. Immune cells that get across the BBB cause neuroinflammation to develop and become chronic. Oxidative stress that is produced as a result of the formation of ROS in the area of inflammation is one of the primary elements contributing to an increase in inflammation. There is proof that oxidative stress plays a significant role in MS aetiology. Therefore, it is known that macrophages and microglia create ROS when they phagocytose myelin in the white matter. The oxidative marker of DNA damage, 8-hydroxydeoxyguanosine, and markers for lipid peroxidation and protein peroxides (protein carbonyls) are additional indicators of the progression of oxidative stress in demyelinating lesions that are inflammatory in MS. Due to mitochondrial malfunction brought on by oxidative damage to mitochondrial DNA and impairment of the function of mitochondrial enzyme complexes in MS lesions, the process of oxidative phosphorylation is interrupted and ROS production by mitochondria rises [151]. Because of this, neurons and glial cells already experience oxidative stress, which causes harm to intracellular proteins, lipids, and DNA as well as the formation of secondary metabolites that may act as additional autoantigens. Additionally, the myelin sheath is directly harmed by ROS, which encourages the production of additional autoantigenic particles, increasing autoimmune inflammation, and subsequently harming neuronal structures. Since oligodendrocytes produce the myelin sheath, their destruction is one of the main contributing elements to the development of MS. Due to lower antioxidant defence, oligodendrocytes are more vulnerable to oxidative damage than neurons and astrocytes, making them an easy target for ROS damage and subsequent axonal demyelination [146, 152]. Recent research indicates that damaged mitochondria are critical to the development of MS. The precise indicators of mitochondrial dysfunction and their correlation with the progression of the disease in various MS types are listed in Table 3 knowing these mechanisms could help develop more focused treatments with better patient results.

**Table 3:** Summary of mitochondrial involvement in multiple sclerosis progression

Tissue	Mitochondria pathology	Cell type	MS phenotype	Reference
Undefined type of tissue	Lower levels of the powerful antioxidant glutathione (GSH) indicate that oxidative stress has a greater impact on the neurodegeneration phase than the neuroinflammation phase	Undefined type of cell	20 PP 20 SP vs 21 RR	[153]
Undefined type of tissue	PGC-1 levels, OXPHOS subunits, antioxidants, and uncoupling proteins 4 and 5 all decreased	Pyramidal neurons	7 PP 7 SP 1 ND 9 C	[154]
Undefined type of tissue	Differing mass spectrometry signals for human cytochrome c oxidase subunit 5b (COX5b), the brain-specific creatine kinase isoform, and the haemoglobin -chain	Undefined type of cell	8 SP 8 C	[155]
Undefined type of tissue	Compared with remyelinating and myelinated axons, greater mitochondrial content and complex IV activity	Acute and chronic demyelinated axons	2 PP 7 SP 1 RC	[156]
Undefined type of tissue	Accumulation of significant mtDNA deletions, some of which exhibit particular loss in complex IV subunits	Neurons	13 SP 10 C	[157]
Active and chronic lesions	Increased amounts of mtHSP70, a heat shock protein that serves as a measure of mitochondrial stress, increased numbers of mitochondria, and mitochondrial protein translation	Undefined type of cell	5 PP 14 SP 7 ND 7 C	[158]
Grey matter in the Cortex	Epigenetic modifications brought on by ROS via decreased activity of NRF-2, a transcription factor for ETC proteins	Undefined type of cell	8 SP 5 C	[159]
Chronic inactive lesions	Increased complex IV activity and overall mitochondrial content	Demyelinated axons	1 PP 9 SP 6 C	[160]
motor cortex	a reduction in the expression of the mitochondrial nuclear gene DNA complex I and III activities that have been functionally diminished	Neurons	1 PP 9 SP 8 C	[161]

In both the acute and chronic stages of MS, mitochondrial dysfunction has a variety of pathways via which mitochondrial impairment advances axonal destruction. Further investigation is done into the effects of mitochondrial dysfunction on more general neurodegenerative disorders. Macrophages and activated microglia produce too much ROS and NO during the inflammatory phase of MS, which causes mitochondrial dysfunction in both myelinated and demyelinated axons. With a major fraction of axons eventually degenerating, intra-axonal mitochondrial swelling in healthy axons is a warning sign of axonal mitochondrial malfunction [139].

**Fig. 5:**
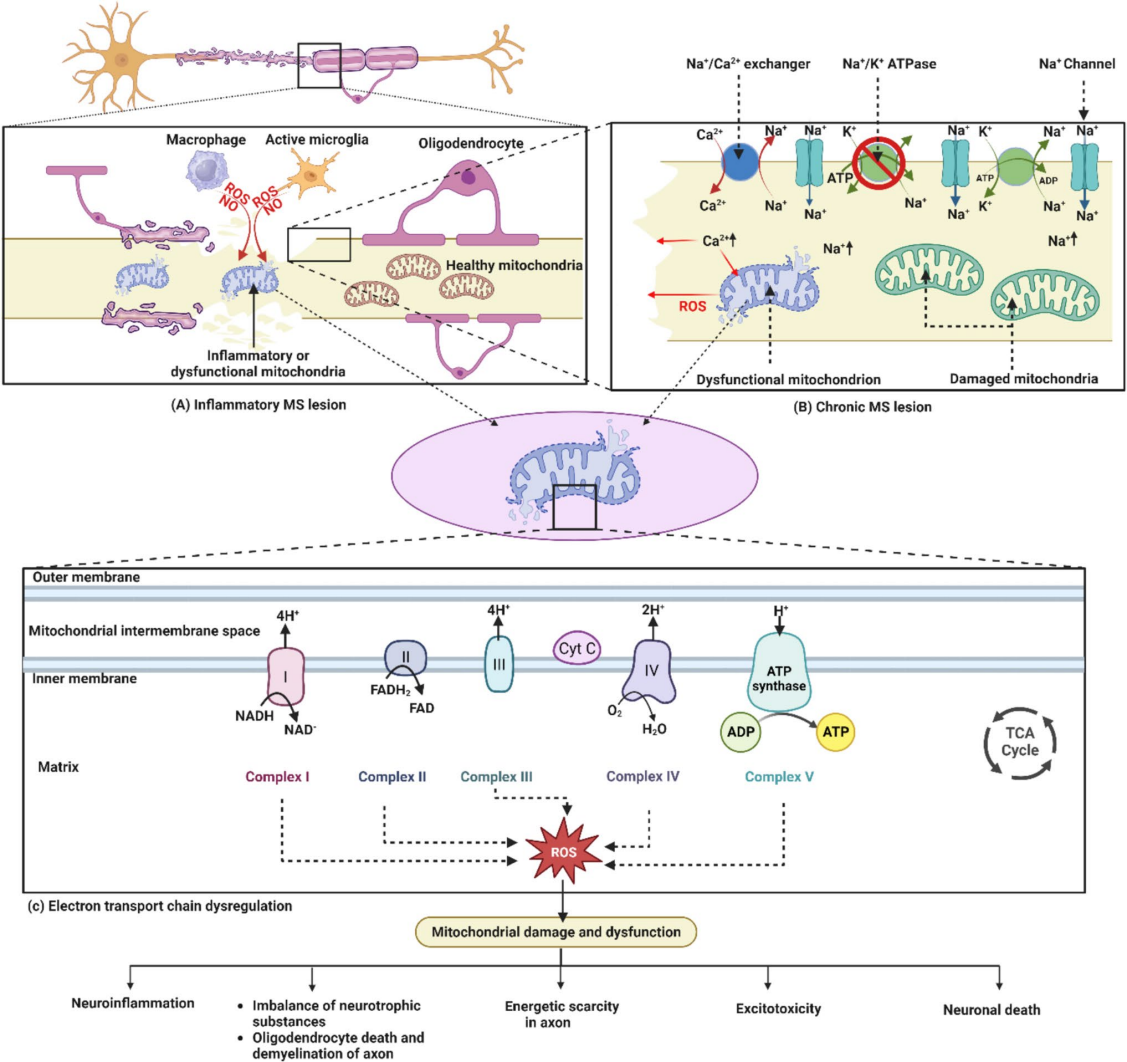
An illustration highlighting the relationship between mitochondrial dysfunction and neurodegenerative pathologies. **A** Inflammatory phase, **B** chronic phase, and (**C**) electron transport chain dysfunction

Axons exhibit more and differently distributed axolemmal Na + channels throughout the chronic phase of MS, raising the intra-axonal Na + concentration. As a result, Na + /K + ATPase has higher energy needs, which raises the amount of mitochondria in chronically demyelinated axons. However, a portion of the mitochondria in these axons are damaged as a result of previous cortical disease or inflammatory damage. Na + /K + ATPase is unable to remove excess intra-axonal Na + as a result, which sets off a chain of events that results in the reversal of the axolemmal Na + /Ca2 + exchanger. This reversal causes increasing axonal Ca2 + concentrations, starting a chain of negative consequences that eventually contribute to axonal degeneration as seen in Fig. 5. These negative effects include further destabilising intra-axonal mitochondria and increased ROS production [162].

Numerous neurodegenerative diseases, characterised by defects in the electron transport chain (ETC) and ATP-synthesis machinery, are thought to be caused by mitochondrial malfunction. The damage is made worse by the release of proapoptotic substances from the mitochondria and ROS produced during the electron transport mechanism. Increased ROS generation, brought on by structural anomalies, damages lipids, proteins, and DNA, especially mitochondrial DNA (mtDNA), in cells. The significance of mitochondrial failure in neurodegenerative illnesses is highlighted by the link between defective mitochondria and ageing and a variety of chronic diseases [163].

Understanding the complex interaction between mitochondrial dysfunction and axonal degeneration in MS improves our understanding of the disease process and creates opportunities for potential therapeutic interventions to slow down the related neurodegenerative processes. The urgent need for focused therapies aiming at maintaining and improving mitochondrial health and function is further highlighted by understanding the larger consequences of mitochondrial malfunction in the setting of numerous neurodegenerative diseases. The pathogenesis of MS is heavily influenced by mitochondrial dysfunction, which causes oxidative stress, neuronal damage, and inflammatory reactions. To effectively manage and eventually treat MS, precise and potent therapy approaches must be developed. To do this, it is essential to have a thorough understanding of the interactions between mitochondrial function, epigenetics, and autoimmune reactions. Further investigation into these complex networks is essential to pinpoint precise targets and strategies for treating the pathology of MS associated with mitochondrial dysfunction. With the use of this understanding, innovative therapies that focus on mitochondrial health will be developed, addressing the fundamental mechanisms underlying the progression of MS and other associated neurodegenerative illnesses. The quality of life for those with MS and similar disorders could be considerably improved with such a focused and nuanced approach, providing hope for future treatments that are more efficient and long-lasting.

## MS Treatment Approaches for Multiple Sclerosis

A neurological condition with many facets, MS has a diverse and complicated pathogenesis. Recent scientific developments have illuminated the critical roles played by mitochondrial dysfunction and epigenetic alterations in the onset and progression of MS. It is crucial to recognise and understand these mechanisms because doing so might lead to creative treatment approaches that could fundamentally alter how MS is managed [8]. Here, we stress how crucial it is to comprehend these pathways to develop viable therapeutic strategies: personalised and targeted therapies [164], new therapeutic targets [165], integrated treatment strategies [166], combating treatment obstacles [167], defining new disease progression indicators [48], and combining traditional and natural therapies.

### Exploration of Various Herbal Treatment Approaches Targeting Epigenetic Modifications and Mitochondrial Dysfunction

When treating MS, a number of natural herbs and the phytoconstituents in them have demonstrated promise in addressing mitochondrial and epigenetic dysfunction. By activating SIRT1, a critical enzyme that promotes mitochondrial biogenesis and lowers oxidative stress, resveratrol contributes significantly to epigenetic regulation and mitochondrial protection. Resveratrol is a good option for MS treatment because of the way hSlps increase mitochondrial function, boost remyelination, and reduce neuroinflammation [168].

Curcumin is another powerful constituent that modulates histone deacetylase (HDAC) activity and inhibits pro-inflammatory pathways to alter epigenetic changes, namely DNA methylation. Two important pathogenic aspects of MS therapy are neurodegeneration prevention and inflammation reduction [169]. Similar to this, the green tea polyphenol epigallocatechin-3-gallate (EGCG) inhibits HDACs and preserves mitochondrial function to control histone acetylation and mitochondrial activity. Its neuroprotective benefits stem from its ability to lower neuroinflammation and oxidative stress, two factors crucial to the development of MS [170]. Flavonoid quercetin is well-known for its capacity to control mitochondrial and epigenetic processes by lowering the production of ROS and blocking DNA methylation. This results in reduced oxidative stress and improved neuronal protection, highlighting its importance in the management of multiple sclerosis [171, 172]. Omega-3 fatty acids also contribute to epigenetic control and mitochondrial biogenesis, modifying DNA methylation and decreasing neuroinflammatory pathways. They are helpful for MS patients because they have the ability to reduce neuroinflammation and improve mitochondrial function [173]. All things considered, natural herbs that target mitochondrial dysfunction and epigenetic alterations, such as resveratrol, curcumin, EGCG, quercetin, and omega-3 fatty acids, show potential in treating MS. By promoting mitochondrial biogenesis, modulating DNA methylation, and activating pathways like SIRT1, these substances aid in the reduction of oxidative stress, neuroinflammation, and neuroprotection. These phytoconstituents provide a good substitute or addition to conventional MS treatments, as the evidence for their advantages grows; nonetheless, more studies are required to optimise their therapeutic application.

### Diet and Exercise Interventions to Manage MS Symptoms and Improve Mitochondrial Function

#### Diet

Innumerable factors that are responsible for the development of MS involve DNA damage, oxidative stress, mitochondrial malfunction, genetic variability, and epigenetic modifications. Numerous studies have demonstrated the positive effects of regular exercise on cognitive abilities and brain health. Furthermore, it is clear how crucial a balanced diet rich in macro- and micronutrients is in halting neurodegeneration and delaying the onset of severe illness [174]. An unhealthy diet such as overindulging in food, a high calorie/a diet low in dietary fiber or low in antioxidants leads to the development of MS, diets rich in antioxidants, anti-inflammatory components, and low-calorie intake are found to decrease age-related cognitive decline and risk of MS [175, 176]. Major sources of plant-derived antioxidants are carotenoids (xanthophylls and carotenes), vitamins (vitamins E and C), fruits, vegetables, drinks, green tea, coffee, spices, nuts, and cereal goods; polyphenols (phenolic acids, flavonoids, anthocyanins, lignans, and stilbenes) proved to have a protective effect in neurodegenerative diseases [177]. A healthy diet such as the Mediterranean diet (MeD) and ketogenic diet can improve the metabolic and lipid profiles in multiple sclerosis. Mediterranean diets are low in red and processed meats and high in fruits, vegetables, legumes, nuts, and whole grains, a moderate intake of fish and red wine should only be moderately consumed with meals, and olive oil is used as the primary source of fat in Mediterranean diets [178, 179]. This diet’s nutritional value suggests consuming vitamins, antioxidants, trace minerals, and polyunsaturated fatty acids (PUFAs), especially ω−3 PUFA; long-term (above 4–6 years) adherence to the MeD has been linked to improvements in cognition, memory, and executive function as well as neuronal integrity, i.e. enhanced cortical thickness and brain volume, reduces the pace of amyloid buildup and hippocampus atrophy, and enhances structural connections [180]. MeD is rich in polyphenols which limit the production of free radicals and also decrease the production of mitochondrial ROS and improve mitochondrial damage, apoptosis, and overall mitochondria function. Components of MeD such as chlorogenic acid, delphinidin (flavonoids), and lycopene (tomato, carotenoid) exerts mitochondria-mediated effects to improve mitochondria functioning [181]. For example, chlorogenic acid increases AMPK, PGC-1 expression, and SIRT1 activity to promote mitochondria biogenesis and reduce ROS [182]. Delphinidin acts by up-regulating nuclear-related factor 1 (NRF1), transcription factor B2 of the mitochondria (TFB2m), transcription factor A of the mitochondria (TFAm), and DNA polymerase gamma (PolG); these help in mitochondrial biogenesis and by restoring the increased levels of mitochondria respiration, mitochondrial DNA content, and complex IV activity [183]; lycopene enhances the expression of silent mating-type information regulation 2 homolog-1 (SIRT1), PGC1α, Cox5b, Cox7al, Cox8b, and cytochrome *c* somatic (Cycs) and poses anti-inflammatory effect, thus improving mitochondrial function [184]. In various studies ketogenic diet (KD) has been found responsible for the reduction of the amount of ROS, regeneration of mitochondria, repairing of myelin sheath of the neurons, reduction in amyloid plaques, the regulation of intestinal microbiota, enhancement of the conversion of glutamine into GABA. Ketogenic diet mimics the metabolic state of starvation, forcing the body to utilise fat as its primary source of energy. The ketogenic diet is recommended for the reconstruction and repair of the myelin sheath [185]. The major brain-derived neurotrophic growth factor (BDNF) produced by neurons involved in myelin repair is affected by the ketogenic diet [186]. The diet works by penetrating the blood–brain barrier with β-hydroxybutyrate, a ketone body, and by influencing NF-KB and mitochondrial respiration, which in turn increases BDNF synthesis indirectly by activating p300/EP300 histone acetyltransferase [187]. Additionally, the ketogenic diet may enhance mitochondrial function, which is critical for cells to produce energy and may be compromised in MS patients [185]. Research indicates that the ketogenic diet promotes oligodendrocyte precursor regeneration and axon remyelination and also decreases the symptoms of autoimmunisation and consequently multiple sclerosis symptoms [188]. The components of the Mediterranean diet/ketogenic diet may improve mitochondrial activity and lower the chance of developing multiple sclerosis. To shed further light on the role of the diet in the pathophysiology of MS, future intervention studies are required to better explore the effects of the ketogenic and Mediterranean diets on mitochondrial function.

#### Excersise

MS can result in a decrease in physical activity possibly due to muscle weakness, abnormal walking, problems in balancing, fatigue, cognitive impairment, etc. Exercise can reverse the impairments caused due to deconditioning but cannot reverse deficits coming from the illness process itself [189]. As we know mitochondrial damage aids in the development of MS, then the treatment approach focused on mitochondrial improvement can play an important role. Mitochondrial biogenesis is regulated by many signalling pathways; among those is the transcription coactivator peroxisome proliferator-activated receptor ϒ co-activator 1α (PGC-1α), which is the main regulator [190]. Acute exercise activates multiple signals, including calcium/calmodulin-dependent protein kinase (CaMK), p38 mitogen-activated protein kinase (p38 MAPK), and AMP-activated protein kinase (AMPK), which converge on PGC-1α (204). Upon activation, PGC-1α interacts with TFs like NRF-1/2, leading to the expression of Tfam, which is imported into mitochondria and crucial for upregulating mtDNA-derived protein transcription [191]. Prolonged exercise lowers ROS generation, indicating improved electron transport capability across the ETC [192]. Endurance exercise results in an enhancement in total mitochondrial proteins, involved in β-oxidation, TCA cycle, and ETC. This whole change improves the capacity for energy supply to the exercising muscle; mitochondria volume increases up to 40–50% with prolonged endurance training. Endurance exercise training improves skeletal muscle aerobic capacity through enhanced mitochondrial quality control, content, and function, but alternative exercise modalities can achieve similar improvements in a shorter period [193]. In the brain, exercise promotes improved mitochondrial function through enhanced complex I activity [194], increased levels of mitochondrial enzymes, and enhanced electron transport chain (ETC) activity and also stimulates the synthesis and secretion of neurotrophins, such as brain-derived neurotrophic factor (BDNF), which can support mitochondrial health in neurons [195]; hence, it is suggested as a potential approach for management of MS symptoms. To provide a comprehensive picture of exercise in MS, a significant amount of investigation in the domains of illness aetiology, activities, and involvement within the ICF framework is required.

### Potential Benefits of Herbal Medicines with Anti-inflammatory and Neuroprotective Effects

Herbal medicines are in great focus of researchers and many herbal medicines are reported to have neuroprotective effects. Sharma et al. (2022) reported the antidepressive-like effect of *Aegle marmelos* leaf extract in chronic unpredictable mild stress-induced depression-like behaviour in rats [24]. Bharadwaj et al. [196] reported that strychnine-induced epilepsy was attenuated by employing *Amaranthus viridis* l. leaves extract in experimental rats [196]. Similarly, many herbal medicines have neuroprotective effects with different mechanisms. The potential benefits of herbal medicines with anti-inflammatory and neuroprotective effects concerning the MS are discussed in Table 4.

**Table 4:** Potential benefits of herbal medicines with anti-inflammatory and neuroprotective effects concerning MS

S. No	Phyto-chemical	Plant	Effect	Benefits	Ref
1	Sinomenine (SIN)	*Sinomenium acutum*	Anti-inflammatory and immunosuppressive	• Inhibits T-bet IFN-gamma pathway to lower iNOS expression, thus have therapeutic application in experimental autoimmune encephalomyelitis • Decrease in axonal injury, demyelination, and inflammatory cell infiltration • The neuroprotective effect is due to Nrf2-dependent anti-oxidative stress and can be used as a potential approach in MS treatment	[197]
2	Embelin	*Embelia ribes*	Neuroprotective effect and anti-inflammatory	• Reduces lipids peroxidation which results in protection from neuronal oxidative damage • Stops the initiation of inflammatory cascades and the synthesis of fibrils linked to amyloid protein	[198]
3	Paeonol	*Paeonia suffruticosa*	Neuroprotective effect and anti-inflammatory	• Have anti-inflammatory and free radical scavenging potential • Reduce the rates of demyelination and leukocyte infiltration, as well as preventing neuronal injury and neuroinflammation	[199]
4	Curcumin	Turmeric plant (*Curcuma longa)*	Anti-inflammatory, anti-oxidant and direct neuroprotective	• Decreases lipid peroxidation, and production of ROS and enhances the activity of SOD and glutathione peroxidase (GSH-px) which results in a decrease in oxidative stress • Enhances expression of Nrf-2 and HO-1 which inhibits neuronal oxidative stress and results in neuroprotection • Decrease T-lymphocyte infiltration in the brain, which could potentially reduce immune-mediated damage in MS	[200]
5	D-Limonene	*Citrus* fruits	Anti-inflammatory and neuroprotective	• Prevent the release of inflammatory mediators, inhibit vascular permeability, reduce neutrophil migration, and display analgesic effects in MS • Increases the amount of IL-10 which inhibits the production of pro-inflammatory cytokine IL-2 thus exerting its anti-inflammatory action	[201]
6	Farnesol	Farnese acacia tree	Anti-oxidant and anti-inflammatory effect	• Due to its ability to block neuronal Ca^2+^ channels, reduce pro-inflammatory cytokine release, and attenuate oxidative stress that leads to therapeutic advantages in experimental MS • Prevent MS patients from CNS inflammatory demyelination by altering the composition of the gut microbiome to a certain extent	[202]
7	Gintonin	Ginseng	Anti-inflammatory	• Can be used in MS treatment due to its anti-inflammatory and anti-oxidant action via LPAR	[203]
8	Olive Leaf tea & Olive Extract	Olive	Neuroprotective and anti-inflammation	• Increases concentration of anti-inflammatory M2 type microglia and preserves myelin and can be effective in MS treatment • Reduce oxidative stress and upregulate SIRT1 expression, which can be an effective approach in the treatment of neurodegenerative diseases	[204]
9	Catechins	Green tea	Anti-inflammatory, neuroprotective, anti-obesity, anti-cancer and Antidiabetic	• Helpful in the treatment of neurodegenerative diseases such as Parkinson’s, Alzheimer’s, and multiple sclerosis • Scavenging of free radicals and chelating metal ions	[205]
10	*Nepeta hindustana* L., *Vitex negundo* L., and *Argemone albiflora* L. Extract	*Nepeta hindustana* L., *Vitex negundo* L., and *Argemone albiflora* L	Anti-inflammatory, anti-oxidative and neuroprotective	• Useful in axonal neuron repair and remyelination • Demonstrated neuroprotective and remyelinating effects, making it a viable natural neurotherapeutic treatment for multiple sclerosis	[206]
12	Β-caryophyllene (BCP)	*Syzygium aromaticum*, *Origanum vulgare* L. and *Piper nigrum* L	Anti-inflammatory, anti-oxidant, and neuroprotective	• BCP can improve the symptoms of animal models of multiple sclerosis at high doses (> 25 mg/kg), i.e. neuroprotective effect • At a low dose (< 10 mg/kg), it gives a better anti-inflammatory effect in the Parkinson’s model	[207]
13	Saffranal	Saffron (*Crocus sativus*)	Anti-inflammatory, anti-oxidant, and Neuroprotective	• Has a role in protecting oligodendrocytes against glutamate and quinoline toxicity, which are associated with multiple sclerosis	[208]
14	Tectorigenin (TEC)	*Pueraria thunbergiana* Benth, *Belamcanda chinensis*, and *Iris unguicularis*	Anti-inflammatory	• TEC inhibits the production of ROS, inflammatory mediators such as PGE2, TNFα, and IL-6 in microglial cells and also decreases activation of NF-kB and C-Jun N-terminal kinase (JNK) proves its potential to be used as a neuroprotective drug in multiple sclerosis and other neurodegenerative diseases	[209]
15	*Terminalia chebula* fruit	*Terminalia chebula*	Anti-inflammation, antibacterial, cardioprotective and Neuroprotective	• Effective in reducing QA (quinolinate) induced cell death, oxidative damage, lipid peroxidation, and oxygen free radicals and protects oligodendrocytes against QA toxicity, thus protecting demyelination, and can be used in neurodegenerative diseases such as multiple sclerosis	[210]
16	Hydroxycitric acid (HCA)	*Garcinia* plant	Anti-inflammatory effect	• By reducing inflammation and oxidative stress, HCA has neuroprotective benefits on nerve damage caused by multiple sclerosis	[211]
17	*Andrographis paniculata* extract	*Andrographis paniculata*	Anti-inflammatory, antifibrotic, and neuroprotective	• In patients with relapsing–remitting multiple sclerosis receiving interferon beta, *A. paniculata* dramatically improves fatigue	[212]
18	Dihydroasparagusic acid	*Asparagus *plants	Anti-inflammatory and Anti-oxidant	• Suppressed microglial activation which is implicated in the inflammatory and oxidative processes leads to the development of neurodegenerative diseases such as multiple sclerosis	[213]

Epigenetic changes and mitochondrial dysfunction are interdependent, which increases their influence on MS pathogenesis. Excess ROS prolongs inflammation and neurodegeneration by causing DNA damage and aberrant methylation. PGC-1α is impacted by impaired histone acetylation, which lowers remyelination and neural energy generation. By blocking antioxidative pathways and raising ROS and oxidative stress, miR-155 worsens mitochondrial dysfunction [17, 18, 26, 116].

## Herbal treatment Approaches for Multiple Sclerosis

A number of plant- and herbal-based remedies are presently being studied for possible MS treatment benefits. Treatments for symptoms including spasticity, inflammation, and neuroprotection, such as osmotin, curcumin, and napiximols, are being evaluated in ongoing clinical trials for their effectiveness. Through methods including regulating cannabinoid receptors, decreasing inflammation, and strengthening cellular resilience against stresses, these medicines show promise in improving patient outcomes and mitigating symptoms associated with multiple sclerosis.

### Summary of Current Clinical Trials Investigating the Effects of Herbal Medicines on MS

Presently, on a global scale, there are a total of 14 clinical trials underway to assess the impact of diverse herbal medicines, dietary interventions, exercise regimens, and therapeutic modalities on the clinical course of multiple sclerosis. Among these clinical trials, seven are directly or indirectly associated with the investigation of herbal medicines or plant-derived extracts. To date among the five, three of these clinical trials have reached completion, two have been prematurely terminated, and two trials are currently in progress or actively recruiting participants. The clinical trials with a primary focus on herbal medicine exploration are engaged towards evaluating the efficacy of nabiximols, osmotin plant protein, curcumin, herbal therapies administered via subcutaneous injection, and DATE-TCM (Traditional Chinese Medicine) in the context of multiple sclerosis, encompassing progressive MS, relapsing–remitting MS, and MS-associated spasticity.

#### Trial to Evaluate the Effect of Nabiximols Oromucosal Spray on Clinical Measures of Spasticity in Participants with Multiple Sclerosis (RELEASE MSS1) [ClinicalTrials.gov ID: NCT04657666]

In a randomised controlled trial, the primary objective is to conduct a comparative analysis of the impact of multiple doses of nabiximols when used as adjunctive therapy, in contrast to placebo, concerning a clinical assessment of velocity-dependent muscle tone in the lower limbs, specifically assessed using the Modified Ashworth Scale Lower Limb Muscle Tone-6 (MAS LLMT-6) [214]. The study focuses on individuals diagnosed with multiple sclerosis (MS) who have not experienced adequate relief from their spasticity symptoms despite prior treatment with other antispasticity medications. The clinical trial was registered on ClinicalTrials.gov with the registration date of November 12, 2020. The study started on December 21, 2020, and was completed on May 10, 2022. The latest update to the trial data was posted on July 20, 2023. A total of 68 participants have been meticulously selected to partake in this randomised, double-blind, placebo-controlled trial, which spans a treatment period of 3 weeks, preceded by a baseline period of 7 days. Throughout the baseline period, participants are instructed to maintain their existing medication regimen and diligently record their spasticity scores. For the duration of their involvement in the trial, participants may be engaged for a maximum period of 90 days, which encompasses a maximum of 29 days dedicated to the screening and eligibility assessment conducted before the commencement of the treatment phase. It is important to note that participants will be advised to gradually escalate the dosage of the investigational medicinal product, be it nabiximols or placebo, over the initial 14 days of the treatment regimen, commencing with a single spray per day and increasing incrementally up to a maximum of 12 sprays per day. The study’s main finding demonstrates that the LLMT-6, a measure of improved muscle tone, is computed by averaging six distinct Modified Ashworth Scale (MAS) transformed scores of the knee flexors, knee extensors, and plantar flexors on both sides of the body. Negative values indicate improvement in muscle tone [214].

#### Herbal Therapy for Subcutaneous Injection Site Reactions in Multiple Sclerosis (MSSkin) [ClinicalTrials.gov ID: NCT00972062]

This research aims to investigate whether the application of an herbal non-prescription cream (0.5 ml) can reduce skin site reactions in individuals with multiple sclerosis who are presently using Betaseron, Copaxone, or Rebif as their subcutaneous treatment for managing their condition. It is worth noting that skin site reactions have been identified as a significant factor leading to the discontinuation of subcutaneous medications (such as Betaseron, Copaxone, and Rebif) for the treatment of multiple sclerosis. The study’s main finding demonstrated that there has been a significant reduction in both the duration and extent of redness, as determined through daily assessments of skin site reactions, which was observed in individuals using the herbal cream in comparison to those using a placebo cream [230,231, ].

#### Dietary Supplement of Curcumin in Subjects with Active Relapsing Multiple Sclerosis Treated With Subcutaneous Interferon Beta 1a [ClinicalTrials.gov ID: NCT01514370]

This investigation constitutes a prospective two-arm, double-blind, placebo-controlled study spanning 42 months. A cohort of 80 participants diagnosed with relapsing multiple sclerosis (MS) has been recruited, with 40 individuals allocated to receive Interferon beta 1a in conjunction with curcumin (BCM95) designated as group A, and the remaining 40 subjects receiving interferon beta 1a paired with a placebo, constituting group B. The *Curcuma longa* plant serves as the source of curcumin, and BCM95, also recognised as biocurcumin, represents a formulation comprising both oil and curcumin extract, thereby enhancing anti-inflammatory, antioxidant, and bioavailability properties in the human system. The study unfolds across an 18-month enrolment phase followed by a subsequent 24-month treatment duration. The study outcomes reveal that group A exhibited a reduced incidence of new lesion growth and a higher proportion of relapse-free patients in comparison to group B [215].

#### Demyelinating Diseases of the Central Nervous System Registry for Patients with Traditional Chinese Medicine (DATE-TCM) [ClinicalTrials.gov ID: NCT05415579]

This observational study is systematically designed with the primary objective of acquiring comprehensive data about the characteristics, safety profile, and efficacy of Traditional Chinese Medicine (TCM) in individuals diagnosed with demyelinating disease of the central nervous system (DDC). The study endeavours to enrol a cohort of 2000 adults within the age range of 18 to 65 years, diagnosed with DDC, over 5 years. The investigative approach involves the collection of baseline data followed by routine follow-up visits scheduled at intervals of 3 to 6 months. These follow-up assessments aim to systematically evaluate the potential long-term safety and efficacy of Traditional Chinese Medicine in the management of DDC, including MS, within the DDC patient population. Furthermore, the study seeks to elucidate the synergistic effects and interaction dynamics between disease-modifying medications and TCM therapy in addressing DDC. The study is still in the recruitment phase and the study is estimated to be completed by 2030 [216].

#### Exploratory Trial of Forza™, a Novel Nutraceutical From Actinidia Deliciosa Plants Bioengineered to Bio-encapsulate the Osmotin Plant Protein as Adjuvant for the Treatment of Progressive Multiple Sclerosis [ClinicalTrials.gov ID: NCT05937802]

This investigation is formulated to assess the anti-inflammatory and neuroprotective efficacy of osmotin, a plant-derived protein marketed under the commercial name “Forza”, in individuals afflicted with progressive multiple sclerosis. The study aims to recruit a cohort of 50 patients, and magnetic resonance imaging (MRI) will be conducted 6 months before treatment initiation, followed by assessments at 1 month and 6 months post-treatment. These assessments will serve to elucidate the impact of osmotin (Forza) on cerebral metabolism and microstructures. The study is currently in the recruitment phase, with an anticipated completion date in 2025 [217].

#### Description of the Selected Herbal Drugs Under Investigation and Their Proposed Mechanisms of Action

The current update regarding the selected herbal drugs under investigation and their proposed mechanisms of action concerning the MS is given in Table 5.

**Table 5:** Current update regarding the selected herbal drugs under investigation and their proposed mechanisms of action concerning multiple sclerosis

S. No	Title	Drug	Description	M.O.A	Clinical trial ID	Ref
1	Trial to Evaluate the Effect of Nabiximols Oromucosal Spray on Clinical Measures of Spasticity in Participants With Multiple Sclerosis (RELEASE MSS1)	Nabiximols	Nabiximol is a herbal medicine formulated from cannabis plant extract, major constituents are delta-9-tetrahydrocannabionol (THC) and Cannabidiol (CBD)	THC has weak partial agonist action at Cannabinoid-1 (CB1R) & Cannabinoid-2 (CB2R) receptors while CBD functions as a negative allosteric modulator of the CB1 receptor	NCT04657666	[214, 218]
2	Herbal Therapy for Subcutaneous Injection Site Reactions in Multiple Sclerosis (MSSkin)	Bach's Rescue Remedy Cream	It’s a combination of 5 different Bach flower remedies (Star of Bethlehem, Rock rose, Clematis, Impatiens & Cherry Plum)	Hydrate and restore the skin to its natural condition	NCT00972062	[219, 220]
3	Dietary Supplement of Curcumin in Subjects with Active Relapsing Multiple Sclerosis Treated With Subcutaneous Interferon Beta 1a	Curcumin (BCM95) & Interferon beta 1a	Curcumin is obtained from the plant *Curcuma longa* (turmeric) majorly found in South Asian countries, especially India	• Curcumin has anti-inflammatory and anti-carcinogenic properties and exerts its action by inhibiting arachidonic acid metabolism, COX, LOX, TNFα, etc • Interferon beta 1a exerts its action by binding to type I interferon receptors (IFNAR1 and IFNAR2c)	NCT01514370	[215, 221]
4	Demyelinating Diseases of the Central Nervous System Registry for Patients With Traditional Chinese Medicine (DATE-TCM)	Traditional Chinese medicine (TCM)	Aims to understand the characteristics, safety, and effectiveness of Traditional Chinese Medicine (TCM) treatment for patients with (DDC) and its interaction with disease-modifying therapy	Under investigation	NCT05415579	[216]
5	Exploratory Trial of Forza™, a Novel Nutraceutical From Actinidia Deliciosa Plants Bioengineered to Bio-encapsulate the Osmotin Plant Protein as Adjuvant for the Treatment of Progressive Multiple Sclerosis	Osmotin (Forza)	Osmotin is a plant protein found in various fruits and vegetables, commercially it is known by the name “Forza”. It helps plants in tolerating both biotic and abiotic stress	• Osmotin has the potential to protect the plant from invading pathogens because of its ability to destroy the plasma membrane of pathogens • Osmotin aids in the build-up of proline, an osmolyte that douse free radicals and reactive oxygen species under stressful situations	NCT05937802	[217]

## Clinical Trial: Investigating Novel Drug Targets in Multiple Sclerosis

The primary aim of disease-modifying therapies (DMTs) activity is to decrease early clinical and subclinical disease activity that can lead to long-term impairment 237. There are now 20 DMTs for MS that are authorised in the USA. All things considered, these drugs work through a variety of neurological pathways to target different aspects of the immune-mediated illness process. The DMTs that are now available and have been approved in the USA are compiled in Table 6.

**Table 6:** DMTs that are now available and have been approved in the USA

Category	DMTs	Pivotal clinical trial	Route of administration	Adverse effects	Reference
Interferons and glatiramer acetate	PEGylated IFN-β1α	In contrast, the placebo ARRR was reduced by 27% for MRI disease activity Disability progression (EDSS): reduced by 38%; GdE lesions: reduced by 86%; T2 lesions: reduced by 67%	Subcutaneous: 125 μg every 14 days	Minor: headache, transaminitis, depression, flu-like symptoms, and injection site responses Serious: thoughts of suicide, anaphylaxis, liver damage, deteriorating rheumatological disorders, congestive heart failure, blood dyscrasias, convulsions, and autoimmune hepatitis	[222]
	IFN-β1α	22 μg low dose three times a week In contrast, placebo ARR was √29%, MRI disease activity was reduced 22%, T2 lesions were reduced 67%, and disability progression (EDSS) was reduced 32% Elevated dosage 44 μg thrice a week In contrast, placebo ARR was approximately 32%, MRI disease activity was 67% for GdE lesions, 78% for T2 lesions, and 28% for disability progression (EDSS) 30 μg once a week Comparison: reduced 18% for placebo; reduced 32% for MRI disease activity; reduced 34% for T2 lesions; reduced 36% for disability progression (EDSS)	Subcutaneous: three times a week, 22 or 44 μg Intramuscular: 30 µg once a week	Minor: headache, transaminitis, depression, flu-like symptoms, and injection site responses Serious: thoughts of suicide, anaphylaxis, liver damage, deteriorating rheumatological disorders, congestive heart failure, blood dyscrasias, convulsions, and autoimmune hepatitis	[223, 224]
	IFN-β1b	Comparatively, the placebo ARR was 34% lower MRI-related disease activity Lesions of GdE: 83% fewer T2 lesions: 75% fewer Reduction of 29% in disability progression (EDSS) (minor variation from the start)	Subcutaneous: every other day, 0.25 mg	Minor: headache, transaminitis, sadness, flu-like symptoms Serious: thoughts of suicide, anaphylaxis, liver damage, deteriorating rheumatological disorders, congestive heart failure, blood dyscrasias, convulsions, and autoimmune hepatitis	[225]
	Glatiramer acetate	Comparative analysis: 29% decrease in placebo-induced ARR; inadequate assessment of MRI disease activity Disability progression according to EDSS: Not noteworthy	Subcutaneous: 40 mg three times a week or 20 mg once a day	Minimal: vasodilator reaction after injection and injection site reactions Major: anaphylaxis, necrosis of the skin, lipoatrophy	[226]
Sphingosine-1-phosphate receptor modulator	Ponesimod	Comparatively, teriflunomide’s ARR was 31% lower When distinct active lesions are merged, MRI disease activity is reduced by 56% EDSS disability progression: decreased by 17%	Oral daily dose titration: 2 mg on days 1 to 2 3 mg on days 3 to 5 4 mg on days 4 to 6 5 mg on days 7 to 8 7 mg on days 9 to 10 9 mg on days 11 to 14 10 mg on days 12 to 14 Maintenance dosage: 20 mg once day starting on day 15 and continuing	Headache, transaminitis, infections, and lymphopenia (absolute lymphocyte count > 200) are minor symptoms Major: macular edema, hypertension, VZV reactivation, PML, lymphopenia (absolute lymphocyte count < 200), bradycardia and bradyarrhythmia upon therapy initiation)	[227]
	Ozanimod	In contrast: IFN-β1a ARR: decreased MRI disease activity by 48% and 38%: Lesions of GdE: 63% and 53% less T2 lesions: 48% and 42% fewer The EDSS disability progression has decreased by 10.8%. The 3-month CDP fell by 8.6%. Six-month CDP	Daily oral dose titration: 0.23 mg on days 1–4, 0.46 mg on days 5–7, 0.92 mg on day 8, and so on Maintenance dosage: once daily at 0.92 mg	Minor: headache, transaminitis, infections (nasopharyngitis, URI), lymphopenia (absolute lymphocyte count > 200) Major: macular edema, hypertension, VZV reactivation, PML, lymphopenia (absolute lymphocyte count < 200), bradycardia and bradyarrhythmia upon therapy initiation	[228, 229]
	Siponimod	Comparatively, the placebo ARR was 55% lower MRI-related disease activity GdE lesions: 82% fewer Lesions in T2: 80% less EDSS disability progression: decreased by 21% 3-month primary end point, or CDP Decreased by 26% 6-month CDP	Oral titration of dose (for daily maintenance of 2 mg): 5 days, 0.25 mg on days 1 and 2, 0.5 mg on day 3, 0.75 mg on day 4, and 1.25 mg on day 5 Dose titration: 4 days, 0.25 mg days 1 and 2, 0.5 mg day 3, 0.75 mg day 4 (for a daily maintenance dose of 1 mg) Maintenance dose: Depending on the CYP2C9 genotype, 1 or 2 mg once daily	Headache, transaminitis, infections, and lymphopenia (absolute lymphocyte count > 200) are minor symptoms Major: macular edema, hypertension, VZV reactivation, PML, lymphopenia (absolute lymphocyte count < 200), bradycardia and bradyarrhythmia upon therapy initiation	[230]
	Fingolimod	Comparatively, the placebo ARR was 54% lower MRI disease activity: 82% reduction in GdE lesions T2 lesions: 75% fewer EDSS disability progression: decreased by 32% Intramuscular IFN-β1a ARR was reduced by 38% (1.25 mg) and 52% (0.5 mg) in comparison MRI disease activity: 0.14 compared. 0.51 (1.25 mg) and 0.23 vs. 0.51 (0.5 mg) for GdE lesions Mean 1.5 versus 2.6 (1.25 mg) and 1.7 vs 2.6 (0.5 mg) were lower in T2 lesions Disability progression: not substantial according to EDSS	0.5 mg taken orally once a day	Headache, transaminitis, infections, and lymphopenia (absolute lymphocyte count > 200) are minor symptoms Major: skin cancer, reactive airway, PRES, macular oedema, heart block, hypertension, herpes, PML, and cryptococcus meningitis; lymphopenia (absolute lymphocyte count < 200); hypertension	[231, 232]
Teriflunomide and cladribine	Cladribine	In contrast: ARR for the placebo was lowered by 58% (3.5 mg/kg) and 55% (5.25 mg/kg) MRI-related disease activity Lesions of GdE: 86% less T2 lesions: 73% fewer Total UAL: fell by 74% EDSS disability progression: decreased by 47% 6-month CDP	The oral cumulative dose of 3.5 mg/kg is split into two treatment courses, each consisting of 1.75 mg/kg; each course is further divided into two treatment cycles spaced out over 2 years	Minor: headache, exhaustion, URI Major: haematological toxicity, lymphopenia, infections, danger of VZV reactivation, cancer, graft-versus-host disease, risk of teratogenicity, and liver toxicity	[233]
	Teriflunomide	7 mg once day Comparatively, the placebo ARR was 31% lower MRI disease activity: 57% reduction in GdE lesions T2 lesions: a 44% decrease 20% decline in disability progression (EDSS) (not significant) 14 mg once a day Comparatively, the placebo ARR was 32% lower MRI disease activity: 80% reduction in GdE lesions T2 lesions: 77% fewer EDSS disability progression: decreased by 26%	7 or 14 mg taken orally once a day	Minor: nausea, thinning hair, and gastrointestinal adverse effects Major: neuropathy, latent TB, teratogenicity (in both sexes), transaminitis, and hypertension	[234]
Fumarates	Dimethyl fumarate	Comparatively, placebo ARR was decreased by 44–53% MRI-related disease activity GdE lesions: reduced by 74 to 90% Lesions in T2: decreased by 71–85% Reduction of 38% in disability progression (EDSS)	Oral Dosage for titration: 120 mg twice a day × 7 days Dosage for maintenance: 240 mg twice a day	Minor: gastrointestinal symptoms, flushing Major: PML, lymphopenia, transaminitis	[235]
	Diroximel fumarate	Temporary information accessible Reduced 77% in GdE lesions with an adjusted ARR of 0.16	Oral Dosage for titration: 231 mg twice a day × 7 days 462 mg twice a day is the maintenance dose	Minor: flushing; less likely to cause gastrointestinal problems than DMF Major: lymphopenia, transaminitis, and PML (presumed risk as DMF); comparable to DMF	[236]
	Monomethyl fumarate	Not available	Oral dose for titration: 95 mg twice a day × 7 days Dosage for maintenance: 190 mg twice a day	Minor: flushing; less likely to cause gastrointestinal problems than DMF Major: lymphopenia, transaminitis, and PML (presumed risk as DMF); comparable to DMF	[237]
Monoclonal antibody therapies	Ofatumumab	In contrast: Teriflunomide ARR: decreased MRI disease activity by 51% and 58% Lesions in GdE: decreased by 97% and 94%; lesions in T2: decreased by 82% and 85% EDSS disability progression: decreased by 34%	Subcutaneous First dosage: 20 mg given in weeks 0 through 2 Next dosage: 20 mg given once a month beginning in week 4	Minor: headaches, lower immunoglobulin levels, injection-related responses, local injection-site reactions, and minor upper respiratory tract infections Major: HBV reactivation, recurring or severe infections	[238]
	Ocrelizumab	In contrast: IFN-β1a ARRR: reduced MRI disease activity by 46% and 47% Lesions in GdE: decreased by 94% and 95%; lesions in T2: decreased by 77% and 83% Progression of a disability EDSS Combined result: a 40% drop	Intravenous First dose: 300 mg IV; second dose: 300 mg IV 2 weeks later 600 mg IV as a maintenance dosage every 6 months	Minor: responses linked to the infusion, minor infections (URI/UTI) Major: serious reactions to the infusion, HBV reactivation, severe or recurring infections, cancer	[239]
	Alemtuzumab	In contrast: IFN-β1a ARR: decreased by 49% and 55% MRI-related disease activity Lesions of GdE: decreased 63% and 61% T2 lesions: decreased by 32% and 17% Disability development (EDSS): declined by 42% and 27% (non-significantly)	Intravenous Initial course: 12 mg IV per day for 5 days Course 2: 12 mg IV every day for 3 days, starting 1 year after the first course Third treatment (if necessary): 12 mg IV per day for 3 days, depending on the course of the illness	Minor: Reactions connected to infusion Major: haemorrhagic and ischemic strokes, hemophagocytic lymphohistiocytosis, infections (HSV, VZV, Listeria), lymphopenia, malignancy, PML, and secondary autoimmune illness (thyroid dysfunction, ITP, Goodpasture syndrome, hepatitis)	[240]
	Natalizumab	In contrast: Placebo ARR: a 68% drop MRI-related disease activity GdE lesions: 92% reduction T2 lesions: 83% fewer EDSS disability progression: decreased by 42%	300 mg intravenously every 4 weeks	Minor: Wearing off phenomena, joint discomfort, headaches, infusion-related symptoms, and exhaustion Major: PML, liver failure, herpes zoster, infections (URI, UTI)	[241]

To lessen long-term disability, DMTs try to decrease early clinical and subclinical disease activity in MS. Although the usage of traditional medications such as interferon-β (IFN-β) and glatiramer acetate (GA) has decreased with the introduction of newer, more effective treatments, they have demonstrated small reductions in recurrence rates and MRI activity. Targeting lymphocyte trafficking, sphingosine-1-phosphate receptor modulators such as fingolimod, siponimod, ozanimod, and ponesimod have demonstrated encouraging outcomes in lowering relapse rates and slowing the advancement of disability. Compared to conventional treatments, these medications have better efficacy and tolerable safety profiles.

In treating MS, this trial attempts to investigate the safety and effectiveness of novel therapeutic targets, and in future, it can with an emphasis on mitochondrial and epigenetic mechanisms. To find novel therapeutic approaches that could supplement current DMTs, interventions focusing on histone changes, DNA methylation, mitochondrial function, and oxidative stress will be assessed. Future studies may employ a randomised, placebo-controlled design to assess the efficacy and safety of therapies targeting the mitochondrial and epigenetic pathways in MS patients. The primary goals will be to reduce annualised relapse rates, disability progression, and MRI markers of disease activity. The goal of the project is to find new therapeutic strategies that target mitochondrial and epigenetic pathways to improve the effectiveness of DMTs now in use. It seeks to shed light on the mechanisms underlying the pathophysiology of MS and open up new options for individualised treatment plans. This trial aims to improve patient outcomes by identifying safe and effective therapeutic approaches and advances our understanding of MS pathogenesis by examining potential pharmacological targets in MS treatment, with a focus on mitochondrial and epigenetic pathways.

Recent clinical investigations concentrating on the impact of herbal medicine on MS management have revealed promising outcomes. Specifically, herbal remedies like nabiximol, curcumin, and a herbal therapy designed for mitigating subcutaneous injection site reactions have demonstrated potential benefits in MS management. Nabiximol has exhibited noteworthy enhancements in muscle tone among MS patients. Studies involving curcumin therapy have indicated a reduced incidence of new lesion growth and a higher proportion of relapse-free patients in the MS treatment cohort. Additionally, the application of herbal therapy, specifically a herbal cream designed for subcutaneous injection site reactions in MS, has demonstrated a significant reduction in both the duration and extent of redness at the skin reaction site. These findings suggest a promising role for herbal medicine in the management of MS, emphasising the need for further research to elucidate the full potential of herbal drugs in MS therapeutic strategies [242]. However, the reported clinical trials have not revealed the epigenetic and mitochondrial pathway, but this could be a great target interest for the researchers to discover a better treatment approach against MS. The understanding of these pathways is important for future discoveries and developments of the potential treatment of MS via epigenetic and mitochondrial dysfunction.

## Discussion

The investigation of herbal medications that target epigenetic changes and mitochondrial dysfunction is a unique and increasingly important strategy to treating MS and could be benificial for other neurodegenerative disorders. MS is an autoimmune condition that affects the CNS and is characterised by persistent inflammation, demyelination, and neurodegeneration, which cause substantial functional deficits. Epigenetic alterations and mitochondrial dysfunction are thought to play important roles in MS pathogenesis, and addressing them provides a potential new therapy option.

The study’s purpose is to investigate how epigenetic alterations, such as DNA methylation, histone modifications, and non-coding RNA control, contribute to MS development. Investigate mitochondrial dysfunction in MS, specifically how decreased energy production, oxidative stress, and apoptosis contribute to neurodegeneration. Identify herbal medications and natural chemicals that can influence these pathways and assess their therapeutic efficacy in MS. Create a basis for future research that incorporates the use of natural substances in clinical practice to treat and maybe reduce the course of MS.

Epigenetic changes, such as DNA methylation, histone modifications, and non-coding RNA changes, affect gene expression without affecting the underlying genetic code. In MS, abnormal epigenetic patterns have been detected in immune cells, notably T and B cells, which play important roles in the autoimmune response that leads to demyelination in the CNS [116]. DNA methylation has been linked to the control of immune response genes, whereas histone changes influence chromatin structure and gene expression. In instance, hypermethylation of the SHP-1 gene has been related to increased inflammation as a result of impaired negative control of pro-inflammatory pathways [243].

MS pathophysiology is characterised by mitochondrial dysfunction, which includes decreased energy generation, increased oxidative stress, and altered calcium homeostasis. Mitochondria are essential for maintaining neuronal health and function, and their malfunction causes ROS buildup and the activation of apoptotic pathways. This leads to neurodegeneration and the breakdown of remyelination mechanisms in MS. Additionally, mitochondrial DNA damage and OXPHOS protein deficiency aggravate the disease’s neurodegenerative characteristics [146].

There is a growing interest in potential treatment approaches for MS include diet, exercise, supplements, and herbal medicines. For example, diets rich in omega-3 fatty acids have been shown to reduce inflammation and may be beneficial in managing MS symptoms. Similarly, exercise has been shown to improve mitochondrial function and may help to mitigate neurodegeneration in MS. Herbal medicines have also shown promise in managing MS symptoms. These herbs have been shown to have anti-inflammatory and neuroprotective effects and may help to address epigenetic modifications and mitochondrial dysfunction in MS. In conclusion, while the exact mechanisms by which epigenetic modifications and mitochondrial dysfunction contribute to MS are still being studied, there is clear evidence to support the involvement of these processes in the disease. Herbal treatment approaches that target these mechanisms may offer a safe and effective way to manage MS symptoms and slow disease progression. Further research is needed to fully understand the potential benefits and limitations of these approaches.

Herbal medicines and bioactive substances have demonstrated the capacity to influence both epigenetic and mitochondrial pathways, allowing for a multi-targeted strategy to treating MS. Several herbal medicines have demonstrated significant promise in targeting both epigenetic alterations and mitochondrial dysfunction, opening up new therapeutic pathways for treating MS. By targeting these two critical elements, natural substances may be able to assist regulate MS's neurodegeneration and autoimmune properties.

Resveratrol, a polyphenol, stimulates SIRT1, an enzyme required for mitochondrial biogenesis and cell stress response. This activation improves mitochondrial function, lowers ROS production, protects mitochondrial DNA, and increases oxidative phosphorylation [244]. Resveratrol’s epigenetic effects include histone deacetylation and re-expression of tumour suppressor genes such as p53, which corrects hypermethylation. Additionally, it boosts PGC-1α, a key regulator of mitochondrial biogenesis [245] Resveratrol’s combined influence on mitochondrial function and epigenetic control presents it as a possible MS therapy. Beyond MS, it has shown efficacy in type 2 diabetes [246] and age-related disorders [247], highlighting its multifaceted function in disease management. Curcumin is widely recognised for its powerful anti-inflammatory effects. It inhibits HDACs, which affects DNA methylation patterns and histone modifications. Curcumin also improves mitochondrial function through antioxidant activity, providing a dual targeting strategy to MS neurodegeneration. Curcumin decreases oxidative stress and neuroinflammation, both of which are important in the pathogenesis of multiple sclerosis [169]. Epigallocatechin-3-gallate (EGCG), the primary polyphenol in green tea, also targets HDACs, avoiding abnormal epigenetic silencing of important genes. EGCG has been shown to inhibit DNA methyltransferases (DNMTs), resulting in the demethylation and reactivation of tumour suppressor genes such as RASSF1A and P16 [248]. This not only has ramifications for cancer, but it also suggests neuroprotective properties in MS. EGCG also decreases mitochondrial ROS and increases ATP production while upregulating mitochondrial biogenesis regulators including TFAM and NRF1. Its capacity to protect neurons from mitochondrial malfunction in neurodegenerative illnesses such as Parkinson’s underscores its therapeutic promise [247]. Quercetin, a flavonoid, influences both epigenetic and mitochondrial functioning by suppressing DNA methylation and lowering ROS levels [249]. Its neuroprotective function in MS is due to its capacity to reduce oxidative stress and promote neuronal survival [250]. Quercetin provides a complete strategy to treating MS by addressing epigenetic silencing as well as mitochondrial dysfunction. Omega-3 fatty acids, which are often present in fish oil, have been shown to modify DNA methylation and reduce neuroinflammation. These fatty acids also stimulate mitochondrial biogenesis and minimise oxidative stress, which helps halt the course of MS. Their capacity to affect both epigenetic and mitochondrial pathways has major implications for controlling MS [251, 252]. Sulforaphane, present in cruciferous vegetables such as broccoli, targets epigenetic changes by blocking HDACs, namely HDAC6. This reactivates suppressed tumour suppressor genes and modifies histone methylation, correcting epigenetic silencing of genes implicated in oxidative stress responses. Sulforaphane also improves mitochondrial function by activating the Nrf2 pathway, which increases antioxidant proteins while decreasing oxidative stress. Its neuroprotective properties in disorders such as Parkinson’s, which stem from its capacity to reduce mitochondrial dysfunction and increase autophagy, suggest that it has the potential to cure MS [253]. Ginkgolides, produced from *Ginkgo biloba*, influence epigenetic processes by modifying DNA methylation and histone acetylation, particularly in neurological illnesses. *Ginkgo biloba* extracts suppress HDACs, giving neurons protection against neurodegeneration. In addition to their epigenetic effects, ginkgolides promote mitochondrial respiration and prevent oxidative damage. Ginkgo extracts have been shown in Alzheimer’s models to inhibit mitochondrial apoptosis, providing insight into its neuroprotective properties, which might be used to MS [254, 255]. Berberine, an alkaloid derived from *Berberis* plants, has demonstrated potential in correcting aberrant DNA methylation in cancer cells and re-expressing tumour suppressor genes such as p53 [256]. It inhibits DNMTs and alters histone modifications, making it an effective epigenetic modulator. Berberine also improves mitochondrial function by activating AMPK, lowering ROS levels, and increasing mitochondrial dynamics [257]. Its significance in metabolic control and mitochondrial function, notably in obesity and diabetes [258], implies that it may have use in neurodegenerative diseases like as MS. These herbal compounds resveratrol, curcumin, EGCG, quercetin, omega-3 fatty acids, sulforaphane, ginkgolides, and berberine target both epigenetic and mitochondrial pathways, making them potential MS treatments. These natural agents modulate DNA methylation, histone acetylation, and mitochondrial biogenesis, providing a multifaceted approach to controlling MS. More study is needed to determine their full therapeutic potential, but their capacity to target the underlying processes of MS represents a fresh approach to treatment. While these herbal substances have showed promise in treating epigenetic and mitochondrial dysfunction in MS, more thorough clinical and preclinical research are needed to confirm their efficacy and find the best dose and delivery strategies. MS’s complexity, including autoimmune and neurodegenerative components, needs a multi-targeted treatment strategy. Herbal medications have a distinct edge in this aspect since they may affect numerous pathways at the same time.

Future study should include in-depth clinical studies to assess the safety and efficacy of herbal treatments for MS; looking at the synergistic impact of mixing herbal substances with established MS medicines to improve treatment results; investigating the molecular mechanisms by which these chemicals influence epigenetic and mitochondrial pathways in greater depth, particularly using sophisticated approaches such as omics and high-throughput screening; and creating innovative drug delivery mechanisms, such as nano-formulations or exosomes, to enhance bioavailability and tailored administration to the CNS.

## Conclusion

Herbal medicines that target epigenetic changes and mitochondrial dysfunction are a promising new therapy option for MS patients. Natural substances such as resveratrol, curcumin, EGCG, quercetin, and omega-3 fatty acids provide multi-targeted treatments that address both neuroinflammation and mitochondrial health, which are two major causes of MS pathology. While these first findings are intriguing, further study is required to fully explore their therapeutic potential. Natural remedies may offer safer and more effective treatment choices for MS.

## Data Availability

No datasets were generated or analysed during the current study.

## Abbreviations

MS: Multiple sclerosis
CNS: Central nervous system
ROS: Reactive oxygen species
ATP: Adenosine triphosphate
EGCG: Epigallocatechin-3-gallate
DNA: Deoxyribonucleic acid
miRNA: MicroRNA
BBB: Blood-brain barrier
OPCs: Oligodendrocyte progenitor cells
HDAC: Histone deacetylase
TCA: Tricarboxylic acid
IMM: Inner mitochondrial membrane
OMM: Outer mitochondrial membrane
ETC: Electron transport chain
NADH: Nicotinamide adenine dinucleotide hydrogen
FADH2: Flavin adenine dinucleotide hydrogen
MMP: Mitochondrial membrane potential
OxPhos: Oxidative phosphorylation chain
mtDNA: Mitochondrial DNA
IHC: Immunohistochemistry
OCR: Oxygen consumption rate
PPMS: Primary progressive multiple sclerosis
RRMS: Relapsing-remitting multiple sclerosis
SAM: S-Adenosyl methionine
TET: Ten-eleven translocation
PAD2: Peptidyl arginine deiminase 2
IFN: Interferon
BDNF: Brain-derived neurotrophic factor
Th: T helper
Tregs: Regulatory T cells
mTORC1: Mechanistic target of rapamycin complex 1
NF-κB: Nuclear factor kappa-light-chain-enhancer of activated B cells
lncRNA: Long non-coding RNA
circRNA: Circular RNA
